# Species relationships within the genus *Vitis* based on molecular and morphological data

**DOI:** 10.1371/journal.pone.0283324

**Published:** 2023-07-31

**Authors:** Jean-Pierre Péros, Amandine Launay, André Peyrière, Gilles Berger, Catherine Roux, Thierry Lacombe, Jean-Michel Boursiquot

**Affiliations:** UMR AGAP Institut, CIRAD, INRAE, Institut Agro, University of Montpellier, Montpellier, France; Institute for Biological Research, University of Belgrade, SERBIA

## Abstract

The grape genus *Vitis* L. includes the domesticated *V*. *vinifera*, which is one of the most important fruit crop, and also close relatives recognized as valuable germplasm resources for improving cultivars. To resolve some standing problems in the species relationships within the *Vitis* genus we analyzed diversity in a set of 90 accessions comprising most of *Vitis* species and some putative hybrids. We discovered single nucleotide polymorphisms (SNPs) in SANGER sequences of twelve loci and genotyped accessions at a larger number of SNPs using a previously developed SNP array. Our phylogenic analyses consistently identified: three clades in North America, one in East Asia, and one in Europe corresponding to *V*. *vinifera*. Using heterozygosity measurement, haplotype reconstruction and chloroplast markers, we identified the hybrids existing within and between clades. The species relationships were better assessed after discarding these hybrids from analyses. We also studied the relationships between phylogeny and morphological traits and found that several traits significantly correlated with the phylogeny. The American clade that includes important species such as *V*. *riparia* and *V*. *rupestris* showed a major divergence with all other clades based on both DNA polymorphisms and morphological traits.

## Introduction

The North temperate *Vitis* genus (*Vitaceae* family) includes a total of 60 to 70 extant species. It is divided into two subgenera, *Muscadinia* and *Vitis* that differ in chromosome number (n = 20 vs n = 19) and several morphological characters [1, 2]. Subgenus *Vitis* includes a single european species *Vitis vinifera* subsp. *vinifera*, which has been domesticated from *V*. *vinifera* subsp. *sylvestris* [3] and is now widely cultivated to produce wine, fruit juice and fresh or dry raisins. The other species are distributed in either eastern Asia or North America [4]. Some of the wild species are cultivated for fruit production for instance *V*. *amurensis* in China and *M*. *rotundifolia* in the United States. Others, such as the American species *V*. *riparia* and *V*. *rupestris*, have been used worldwide as rootstocks. However, the wild species have mostly been used as parents in crosses aiming to improve the resistance to diseases or confer adaptative traits both in scion and rootstocks cultivars [5–7].

Breeding interspecific hybrids is made easier because most *Vitis* species are interfertile. On the other hand, natural and past artificial hybridization could also complicate species delimitation [4, 8]. For instance, based on morphology, ecology and geography, the classifications of subg. *Vitis* by Planchon [9], Munson [10], Bailey [11], Galet [12] and Moore & Wen [2] are not in full agreement for the number of series, the placement of species in series and also for distinguishing hybrids from pure species. Furthermore, these classifications used different synonymous names and apart those of Planchon [9] and Galet [12] mainly concerned the North American species. Chinese *Vitis* species taxonomy has been more recently investigated [8, 13, 14] and also revealed problems of species boundaries and synonymies. These difficulties in the taxonomic treatment when using morphological and anatomical characters have been considered due in part to hybridization and clinal variation [1, 4, 15, 16].

Recent molecular studies allowed clarifyig the classification of subg. *Vitis*. It has been shown that the Eurasian species (including *V*. *vinifera*) and North American species constitute two well-separated clades each containing several subclades [17–28]. The two main clades diverged recently between the middle-late Miocene and the late Pliocene [28] and most studies agree on that partition. However, the species relationships within clades vary according to the analyzed sample, the type of data (i.e. chloroplast vs. nuclear, single genes vs. SNP arrays or NGS data) and the method used for phylogeny reconstruction. Such difficulties may result from the sharing of ancestral polymorphisms due to recent divergence and reticulation due to historical recombination [22]. For this last point, recent studies highlighted hybridization within the Asian clade [29] and within the American one [30]. The misidentification of accessions and the presence of hybrids in the studied samples may add background noise that could result in weakly supported nodes in phylogenetic trees.

Leaf morphology broadly varies among species and varieties in the *Vitis* genus [31]. *Vitis* leaves differ for instance in their degree of lobing, in the lengths and angles of their five major veins and in margin dentation. Ampelographic measurements have therefore been largely used to discriminate between *Vitis vinifera* cultivars [31–36], and more recently *Vitis* species [37–39]. Moreover, Chitwood et al [40] found evidence for an important genetic component determining leaf shape and venation patterning in grape. Therefore, besides their interest for taxonomy, some morphometric measures may be useful in the breeding of new varieties with adaptative traits for the undergoing climatic changes. In a recent work, Ma et al. [41] used a phylogenomic framework within the *Vitis* genus to decipher the evolution of several morphological characters, e.g. tendril architecture, leaf shape, and type of trichomes. They found several examples of convergences, and revealed in the Asian *V*. *bryoniifolia* subclade the presence of cryptic species having similar morphology and distribution.

In this study, we assessed genetic and morphological diversity in the *Vitis* genus throughout its geographic range within a comprehensive sample of species and putative hybrids. Our main objectives were 1) to clarify some standing problems in the *Vitis* phylogeny and 2) to evaluate the importance of the phylogenetic signal in morphological characters in comparison with convergence due to similar environments. Analyses helped to clarify the relationships among species and allowed hybrids identification. Morphological variation was found both within species and among clades and several traits were found autocorrelated with the phylogeny.

## Materials and methods

### Plant material and DNA extraction

A total of 90 *Vitis* accessions maintained at the INRAE repository of Vassal-Montpellier (Marseillan, France, www6.montpellier.inrae.fr/vassal_eng) were used during this study (S1 Table). This set included 13 Asian and 56 North American accessions representing 12 and 18 putative species, respectively. For *Vitis vinifera* we retained a set of eight cultivars (subsp. *vinifera*) amongst the most popular French ones and a set of eight wild accessions (subsp. *sylvestris*) mostly originated from French populations. *Muscadinia rotundifolia* was added as an outgroup with five accessions. Most of this germplasm has been studied and precisely identified [12] but seven accessions (THU2, YES, AES5, AES6, ARZ2, TRE, TRE2, S1 Table) were more recently introduced to increase the number of species or the number of accessions for some species. Total genomic DNA was extracted from young leaf tissues using the DNeasy 96 plant mini kit (Qiagen Inc., Valencia, CA, USA). DNA concentration and purity was assessed using a NanoDrop 8000 UV-Vis spectrophotometer (Thermo Scientific).

### Loci sequencing

Twelve nuclear sequences (S2 Table) were amplified and sequenced in 78 accessions (S1 Table) according to Péros et al [42]. Primers were either already published or specifically designed for this study using Primer3 software [43]. *GAI1* [44] and *TFL1* [45] genes are involved in grape development and *GAI1* has already been used in grape phylogenetics [46]. *LDOX*, *CHI1* and *DFR4* code for key enzymes in the flavonoid biosynthetic pathway in grape [47]. TC1-A and TC1-B are located in a large intron of around 1,500 bp that contains several repeat motifs. As *Vitis* species are highly heterozygous, several steps were needed to design primers to partly sequence this intron. The five other amplicons were selected among a large set of amplicons designed to obtain a SNP-based diversity map in the *Vitis* genus (INRAE project SNPGrapeMap). Polymerase chain reaction (PCR) was performed in 25 μL volumes using the following protocol for all sequences: denaturation at 94°C for 3 min, 35 cycles at 94°C for 30 s, 55°C for 1 min, and 72°C for 1 min 30 s, followed by 6 min elongation at 72°C and 10°C soak. Samples containing 1 μL of PCR products and 10 μl of de-ionized water were run on a capillary sequencer ABI Prism 3700 XL (Applied Biosystems, CA, USA). Chromatograms of the forward and reverse strands were aligned and edited with deletion of indels using the software CLC Sequence Viewer 6.0 (CLC bio then Qiagen) to generate alignment files in fasta format.

### SNP array genotyping

The GrapeReseq 20 K SNPs array [48] was used to genotype 78 accessions (S1 Table) according to Illumina GoldenGate assay protocols [49] at the GenoToul platform (Toulouse, France). DNA from ‘PN40024’ corresponding to the reference genome [50] was included as control. Genotyping was performed using the Genotyping Module v1.9 of the Illumina GenomeStudio Data Analysis software. Manual curation and several filters were applied following Laucou et al [51]. We finally used the genotyping at 12,971 SNPs with only 2.04% of missing data for the 78 accessions. Among these SNPs 83.06%, 14.35% and 2.57% were discovered in *V*. *vinifera*, several American *Vitis* and *M*. *rotundifolia*, respectively (S3 Table).

### Phylogenetic analyses

Chromatograms of forward and reverse strands were aligned and edited using the software CLC Sequence Viewer 6.0 (CLC bio then Qiagen). All positions corresponding to indels in one or several individuals were deleted. Final alignments were generated in fasta format and analyzed separately or after concanetation. Phylogenetic analyses were first conducted using MEGA7 [52]. Other analyses were performed in the R environment 3.6.0 [53] using the following R packages: apex v.1.0.3 [54], phangorn v.2.5.5 [55], ape v.5.3 [56] and adegenet v.2.1.1 [57]. Phased genotypes were obtained using the algorithm GEVIL of GEVALT software [58] implemented within SNIPlay software [59]. Haplotype networks were then constructed using the Median Joining method implemented in PopART software [60].

### Ampelometric measures

Leaves from 70 accessions were collected at the end of spring at Vassal-Montpellier repository. Four to 18 fully expanded leaves per accession (median number = 8.5) at similar developmental stage were taken from the middle of shoots. Leaves were dried in a plant press between newsprint sheets. The abaxial surface of each leaf was photographed using a camera mounted on a copy stand. Parameters were obtained from these pictures using SuperAmpelo ver. 2.0 [61] and corresponded to lengths, distances, angles and calculated values (area, ratio). Median values per accession were considered and averaged for parameters measured on the right and left halves of leaves. In addition, photographs were processed using ImageJ [62] to obtain from binary images measurements of the aspect ratio AR and circularity (Circ = (4π × (area/perimeter^2^) [40]. The 39 parameters were grouped according to three main types of quantitative variables: length and distances (n = 16), angles (n = 10) and ratios (n = 13); they are listed in S4 Table and illustrated in S1 Fig.

### OIV descriptors

Accessions were observed directly in the vineyard (INRAE repository of Vassal-Montpellier) for a total of 46 OIV descriptors [63] assessed at different development stages of leaves and shoots (S5 Table). Observations had already been recorded across several past vegetative seasons and were completed during this study to avoid missing data. These descriptors were grouped according to three types of traits: morphology (n = 15), color (n = 13), and pilosity (n = 18).

### Morphometric analyses

Analyses were performed without hybrid accessions (known or identified) and for accessions having all three types of data (sequence data, leaf measures, OIV codes). This subsample comprised 56 accessions and represented 24 species or subspecies. Leaf measurements and OIV descriptors were analyzed separately using Principal Component Analysis (PCA) implemented in the R package FactoMineR v.1.42 [64]. Results were visualized using the R package factoextra v1.0.5 [65]. We also analyzed the phylogenetic signal (i.e. phylogenetic autocorrelation) present in each data set using the R package adephylo v1.1.11 [66]. The non-parametric Abouheif’s test [67] was used to detect traits with significant autocorrelation based on a randomization test with 999 permutations. Then, a phylogenetic principal component analysis (pPCA) was performed to summarize our sets of traits into a few synthetic variables showing positive (global structure) or negative (local structures) phylogenetic autocorrelation in the phylogenetic tree. This latter was that previously obtained using the alignment of the 12 loci.

## Results

### Species relationships based on sequence data

**Fig 1:**
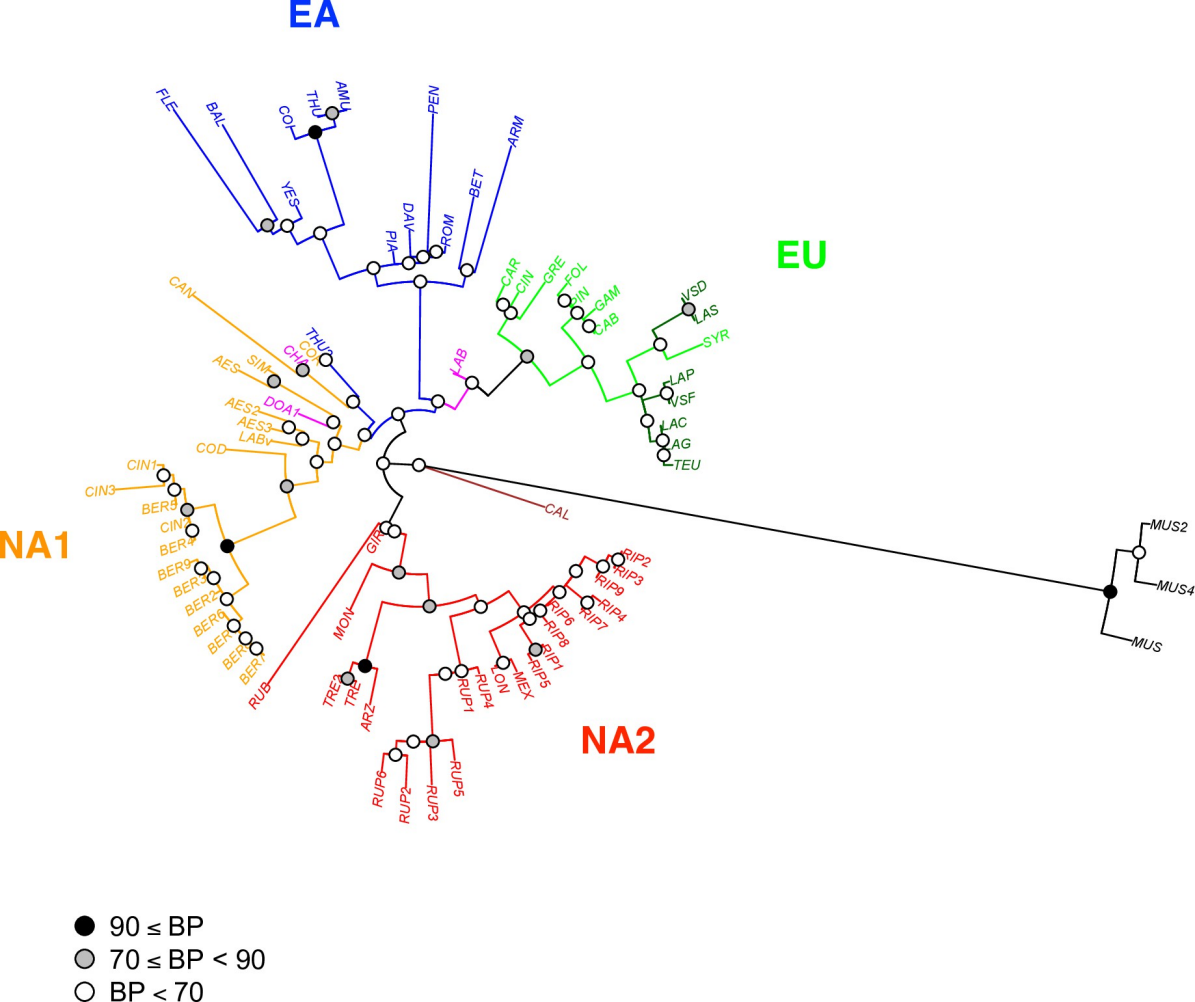
ML tree obtained using nuclear sequences. *Vitis* accessions (codes in S1 Table) were grouped in separated clades: EA (East Asia, blue), EU (Europe, spring green: *V*. *vinifera* subsp. *vinifera*, dark green: *V*. *vinifera* subsp. *sylvestris*), NA1 (North America 1, orange), NA2 (North America 2, red), *V*. *californica* (brown) and *M*. *rotundifolia* (grey). Known hybrids were figured in pink (CHA, DOA1, LAB). The tree is drawn to scale, with branch lengths measured by the number of substitutions per site. The percentage of trees in which the associated accessions clustered together is shown at nodes with threshold at 70 and 90%.

The final aligment for 12 loci and 78 individuals was 6,771 bp in length. MEGA7 detected 434 variables sites including 261 parcimony informative sites and 173 singletons. However, heterozygous data (IUAPC codes: K, M, R, S, W, Y) were not considered by the software and the level of polymorphism was therefore underestimated using the diploid data. The Maximum-Likehood tree with the bootstrap values calculated from 100 replicates were exported in newick format and then edited using package ape in R (Fig 1). The outgroup *Muscadinia* was well separated from the accessions of subgenus *Vitis* and among these latter the American species *V*. *californica* had a separate position. The Eurasian accessions were grouped in two sister clades corresponding to the East Asian species (EA) and to wild and cultivated *V*. *vinifera* (EU). Other North American accessions were distributed in two separate clades: NA1, sister to the Eurasian clades, and NA2. Nodes were generally poorly supported, with the exception of the *V*. *coignetiae*-*V*. *thunbergii-V*. *amurensis* group in the EA clade, the *V*. *cinerea* complex in the NA1 clade and the *V*. *trealesii*-*V*. *arizonica* group in the NA2 clade. Cultivated and wild accessions of *V*. *vinifera* were separated with the exception of cv. Syrah, which grouped with the wild accessions. The position of the hybrid between *V*. *vinifera* and *V*. *labrusca* (LAB, cv. Isabelle) occupied an intermediate position between EU clade and NA1, which contained the pure *V*. *labrusca* species (LABv). By contrast, the other known hybrids *V*. × *champinii* (CHA) and *V*. × *doaniana* (DOA1) were imbedded within the NA1 clade. The second putative accession of the East Asian *V*. *thunbergii* (THU2) occupied a basal position in the NA1 clade suggesting an hybrid parentage.

To assess genetic diversity and identify the hybrids existing in our sample, haplotype sequences were inferred from genotype data for each individual locus. The number of SNPs per locus varied from 29 (*LDOX*) to 106 (*CHI1*) for a total of 674 SNPs (Table 1) indicating that 240 polymorphic positions (674 minus 434) were not taken into account in the ML tree (Fig 1). The number of haplotypes per locus varied from 27 to 66 with similar diversity statistics among loci; in average, ThetaW, Pi and He were 0.0214 (range 0.0118–0.0387), 0.0106 (range 0.0063–0.0177) and 0.92 (range 0.863–0.953), respectively (Table 1). Using a custom R script, we calculated the number of differences between the two haplotypes for each accession. The values averaged over the 12 loci were figured as boxplots for each group identified in the ML tree (S2 Fig). Outliers comprised, as expected, the recognized hybrids *V*. *× labrusca* (LAB), *V*. *× champinii* (CHA) and *V*. *× doaniana* (DOA1) and other outliers might therefore be also suspected to be hybrids: *V*. *coriacea* in the NA1 group, one accession of *V*. *rupestris* and *V*. *girdiana* in the NA2 group, and accessions assigned to *V*. *yeshanensis*, *V*. *thunbergii* and *V*. *piazeskii* in the East Asian group (EA).

**Table 1:** Diversity statistics calculated for phased haplotypes of 12 loci sequenced in a set of *Vitis* accessions.

Locus	N	Size (pb)	Nb SNPs	Nb haplotypes	ThetaW	Pi	He	D Talima	Fay & Wu
*GA1*	77	650	43	34	0.0118	0.0063	0.863	-1.407	4.078
*TFL1*	67	712	66	52	0.0167	0.0082	0.966	-1.603	5.808
*LDOX*	70	260	29	39	0.0202	0.0138	0.925	-0.926	3.592
*CHI1*	78	703	106	53	0.0266	0.0099	0.953	-1.991	6.933
*DFR4*	70	219	34	27	0.0282	0.0111	0.831	-1.796	2.438
*TC1A*	72	312	67	53	0.0387	0.0173	0.953	-1.731	5.385
*TC1B*	73	323	55	44	0.0306	0.0177	0.953	-1.300	5.721
*255A*	75	369	42	28	0.0204	0.0095	0.873	-1.601	3.519
*1526A*	77	526	47	46	0.0159	0.0078	0.946	-1.547	4.099
*2351A*	75	704	52	34	0.0132	0.0075	0.923	-1.318	5.301
*4194A*	77	617	52	38	0.0150	0.0081	0.914	-1.408	4.994
*4275A*	76	758	81	66	0.0191	0.0097	0.961	-1.540	7.371

N, number of individuals in the alignment

**Fig 2:**
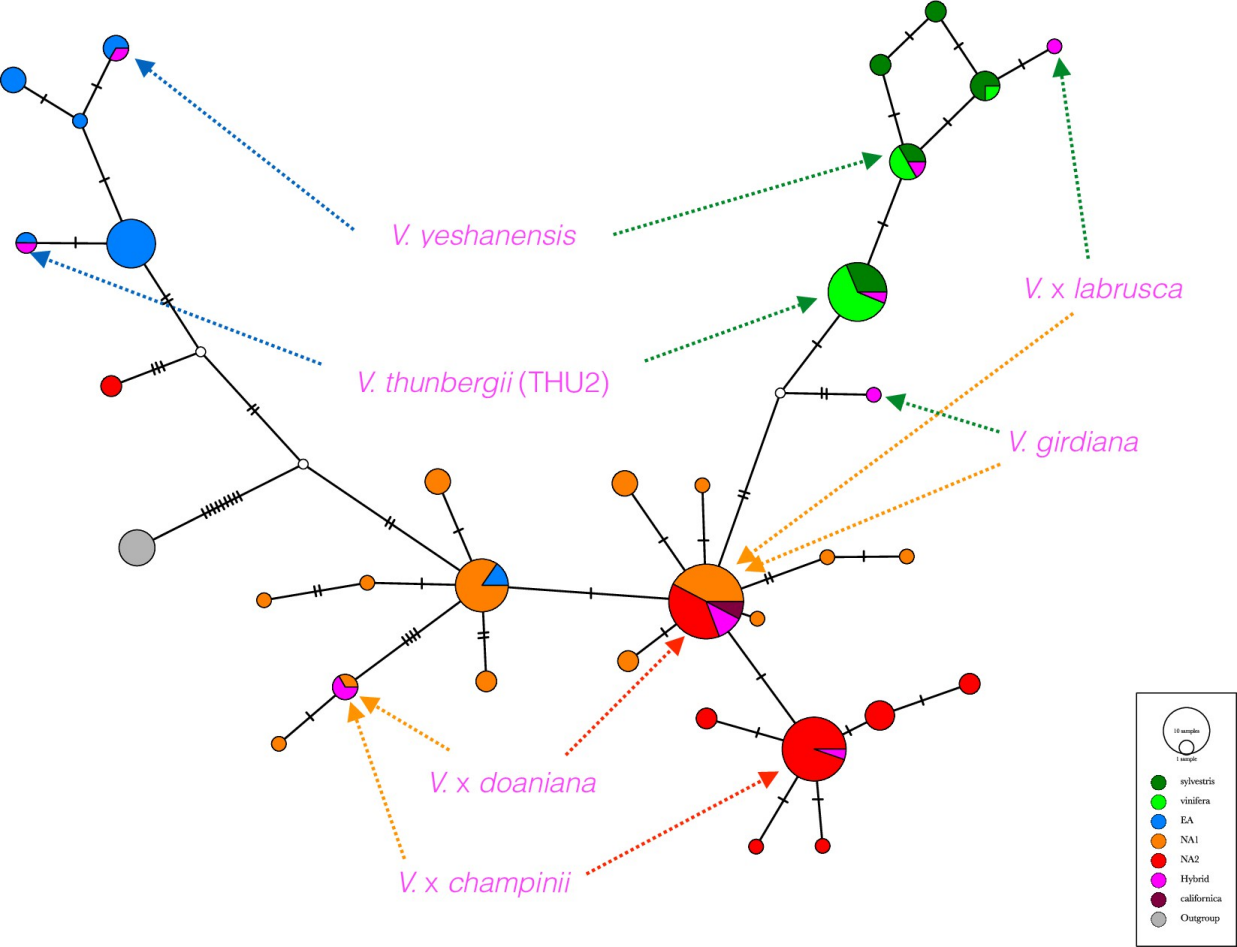
Haplotype network for the 2351A locus. Haplotypes of known (CHA, DOA1, LAB) and putative (GIR, THU2, YES) hybrids (colored in pink) occupied separate position in the network. Other *Vitis* haplotypes were colored as in Fig 1 considering the clade to which they belong.

To clarify the origin of these putative hybrids we analyzed the haplotype networks for each of the 12 loci. From the network obtained with locus 2351A (Fig 2), it clearly appeared that *V*. *girdiana* resulted from hybridization between *V*. *vinifera* and an American species, whereas both *V*. *thunbergii* (THU2) and *V*. *yeshanensis* were hybrids between *V*. *vinifera* and Asian species. The high heterozygosity of *V*. *× labrusca* (cv. Isabelle) was due to a well-documented artificial cross between *V*. *labrusca* and *V*. *vinifera*. High heterozygosity in *V*. *× champinii* and *V × doaniana* resulted from natural hybridization between species belonging to the two distinct American clades NA1 and NA2. Networks obtained for other loci were shown in S3 Fig. Sequences such as *GAI1* (S3A Fig), *CHI1* (S3B Fig) and *4275A* (S3C Fig) indicated that both hybrids shared one haplotype with *V*. *candicans* whereas their second haplotype belonged to an American species of clade NA2, most probably *V*. *rupestris* for *V*. × *champinii* and *V*. *riparia* for *V*. × *doaniana*.

**Fig 3:**
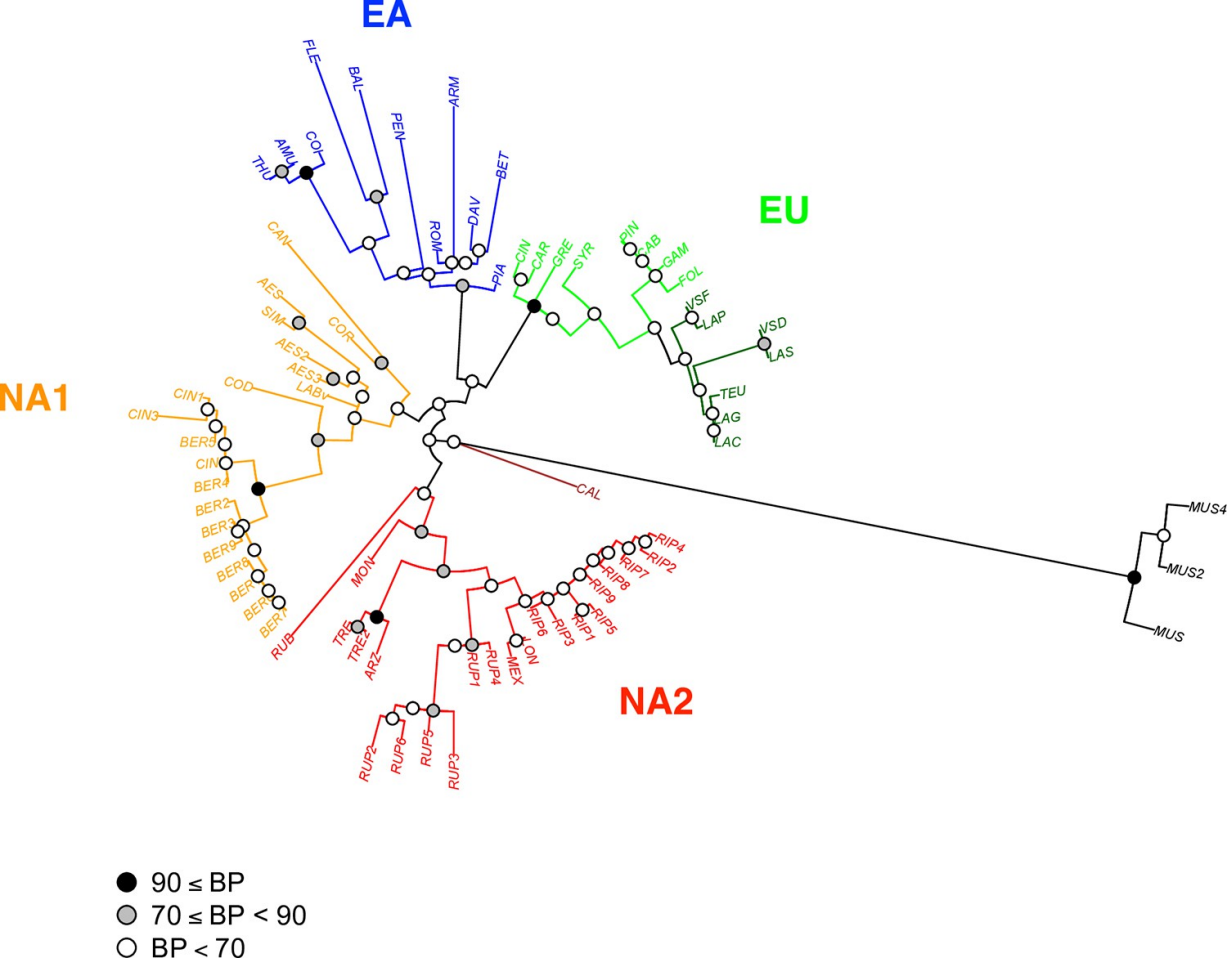
ML tree obtained without hybrids using nuclear sequences. Six *Vitis* hybrids were not included in this analysis (CHA, DOA1, LAB, GIR, THU2, YES). Same legend than Fig 1.

We constructed another ML tree without the known or newly identified hybrid accessions (Fig 3), with the aim to better depict the relationships among pure species. Branch supports were not greatly improved but some changes in accession placement were observed. Within the Eurasian clade, *V*. *piazeskii* (PIA) now occupied a sister position in the EA clade and the two subspecies of *V*. *vinifera* were separated although without strong support. In the NA1 clade, the *V*. *cinerea* var. *cinerea* and var. *berlandieri* accessions constituted a well-separated branch with *V*. *cordifolia* (COD) in a sister position. Another clade was represented by *V*. *aestivalis* and *V*. *labrusca*, whereas *V*. *candicans* (CAN) was a distant species at the base of the NA1 clade. This part of the tree might be obscured by a possible hybridization between *V*. *aestivalis* (AES) and *V*. *candicans* (CAN) either with *V*. *simpsonii* (SIM) or more probably *V*. *coriacea* (COR) as suggested by the high heterozygosity in this latter accession (S2 Fig). The *arizonica*-*rupestris*-*riparia* clade within the NA2 clade also included *V*. *longii* (LON, syn. *V*. *acerifolia*) and *V*. *novo mexicana* (MEX). *V*. *rubra* (RUB, syn. *V*. *palmata*) and *V*. *monticola* (MON) occupied a basal position in this clade. The position of *V*. *californica* was not changed after the exclusion of hybrids and was always clearly separated from other accessions of the subgenus *Vitis*.

### Species relationships based on SNP array

**Fig 4:**
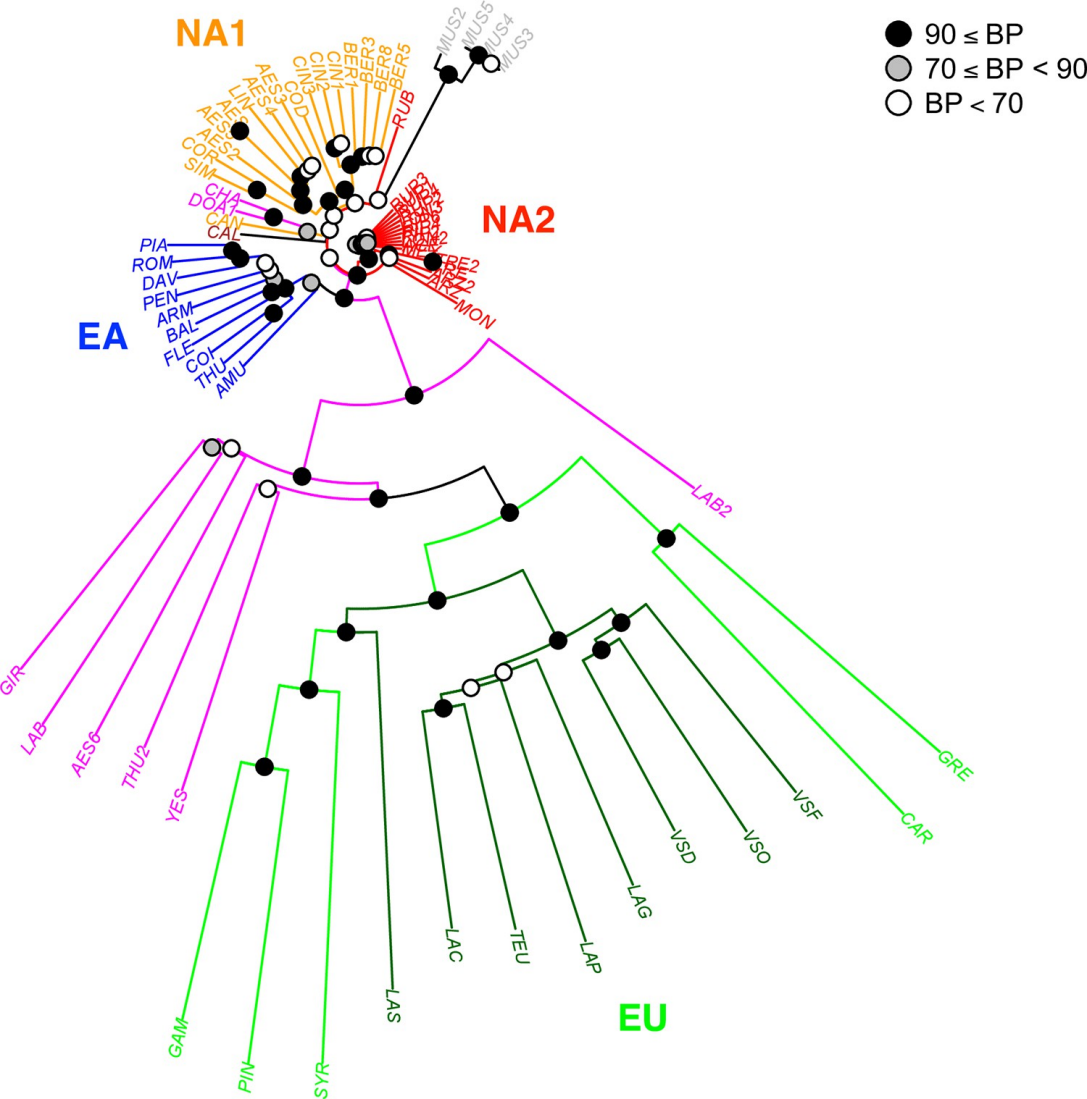
Distance tree constructed using data from the SNP array. Colors used for hybrids and clades as in Fig 1. The percentage of trees in which the associated accessions clustered together is shown at nodes with threshold at 70 and 90%.

The full data set included 70 accessions genotyped at 12,971 SNPs. The distance tree constructed with the NJ algorithm confirmed the accessions placement of in the four clades identified using the sequence data but with a higher number of highly supported nodes (Fig 4). Hybrids that had a *vinifera* parent clearly appeared at the base of the *vinifera* clade. In addition to LAB (cv. Isabelle), GIR, THU2 and YES, they included two accessions without sequence data: another *V × labrusca* hybrid (LAB2, cv. Concord) and a falsely identified putative accession of *V*. *aestivalis* (AES6). Since around 83% of the array SNPs originated in *V*. *vinifera* (S3 Tab), branch lengths were very long for *V*. *vinifera* accessions and hybrids with this species. On the contrary, branch lengths were short in the other clades especially in NA2 that was not represented in the discovery panel. The East Asian clade also was not represented in the discovery panel but some SNPs discovered in *V*. *vinifera* might be expected to exist in the East Asian accessions. As another consequence of this ascertainment bias, the mean observed heterozygosity was high and highly variable in the European clade, but low and less variable in the other clades, especially in NA2. Hybrids with *V*. *vinifera* again corresponded to outliers in the box-plots (S4 Fig), whereas *V*. *× champinii* (CHA) and *V*. *× doaniana* (DOA1), although not identified as outliers, presented the highest values of heterozygosity within the NA1 clade.

**Fig 5:**
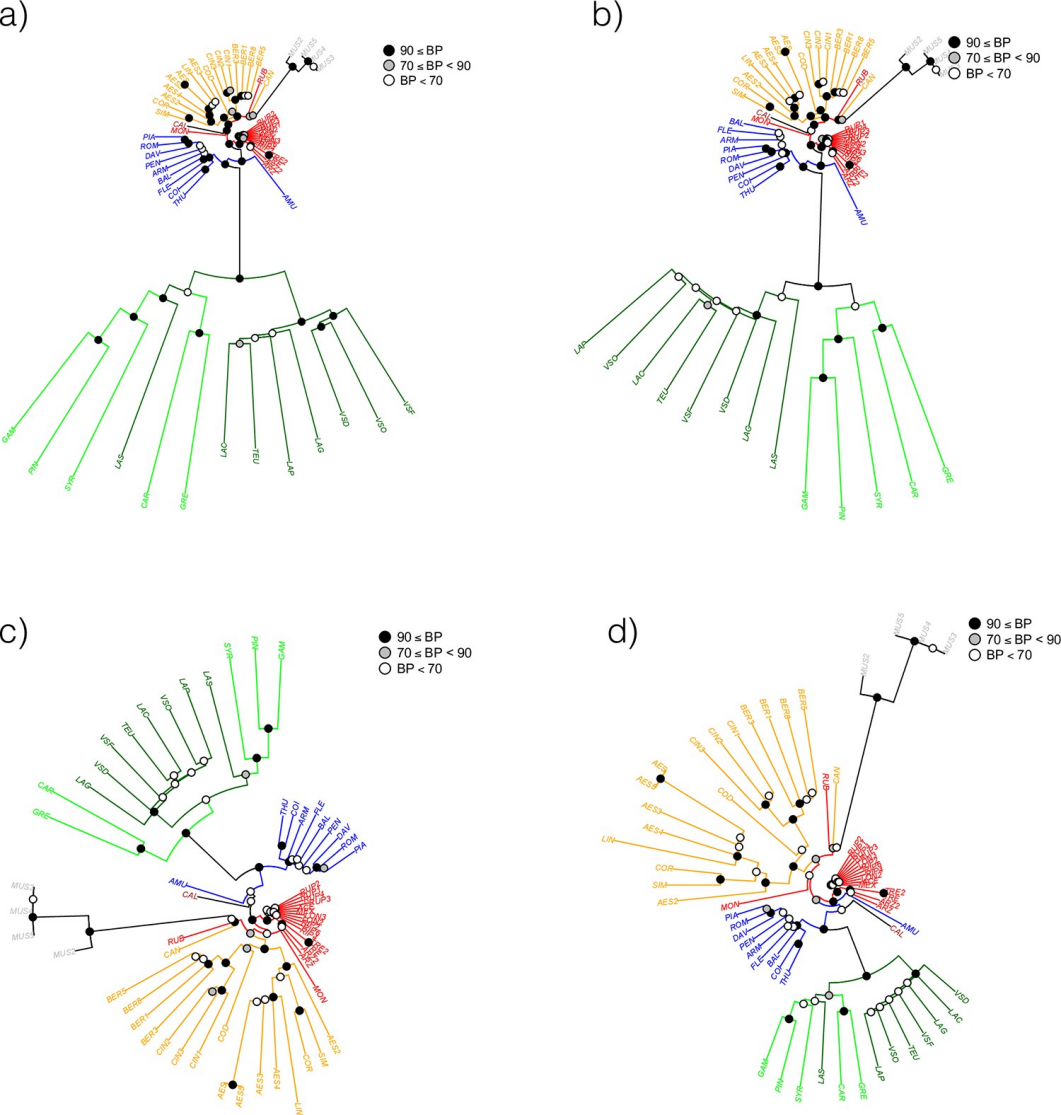
Distance trees constructed with different sets of SNPs from the array. a) 12,971 SNPs with 10,774 *V*. *vinifera* SNPs, b) 7,197 SNPs with 5,000 *V*. *vinifera* SNPs), c) 3,197 SNPs with 1,000 *V*. *vinifera* SNPs, and d) 2,697 SNPs with 500 *V*. *vinifera* SNPs. Hybrids are not included. *Vitis* clades colored as in Fig 1. The percentage of trees in which the associated accessions clustered together is shown at nodes with threshold at 70 and 90%.

We then excluded the hybrids to construct another distance tree (Fig 5A). Differences in branch length were clearly visible but clade arrangement was not evident especially for the species occupying basal positions in the clades. To reduce the effect of ascertainment bias and therefore better visualize the relationships between non-*vinifera* accessions, we constituted three other data set with decreasing fractions of vinifera SNPs sampled at random. The new SNP sets had 5,000 (69.4%), 1,000 (31.3%) and 500 (18.5%) vinifera SNPs, respectively. Decrease in the percentage of *vinifera* SNPs did not change the species relationships but allowed visualizing better the relationships in non-*vinifera* accessions by modifying branch lengths (Fig 5B–5D). *V*. *amurensis* appeared basal in the Eurasian clade (EA+EU). For American taxa, except the clearly differentiated clades such as *aestivalis* or *cinerea*-*berlandieri* in NA1 or *arizonica*-*rupestris*-*riparia* in NA2, the position of *V*. *californica*, *V*. *candicans*, *V*. *rubra* and *V*. *monticola* were not fully congruent with the relationships depicted using sequence data (Fig 3).

### Polymorphim in chloroplast DNA

A total of 23 SNPs in the array corresponded to polymorphisms in chloroplast DNA (Ibanez, pers. comm.). Only 11 of these loci were informative (biallelic) in our sample of 70 accessions and allowed defining 19 chlorotypes with 1 to 8 SNP differences and corresponding to 1 to 16 accessions (S5A Fig). The hybrids THU2 and YES shared their chlorotype with accessions from the European clade and with *V*. *californica*, therefore they issued most probably from a hybridization between an Asian species as female and *V*. *vinifera* as male. The GIR and LAB (cv. Isabelle) hybrids shared their chlorotype with American accessions and therefore *V*. *vinifera* was again the male parent. The CHA and DOA1 hybrids had a chlorotype differing from *V*. *candicans* so their female parent was more probably *V*. *rupestris* for CHA and *V*. *riparia* for DOA1 according to the nuclear results. Using the *mlg*.*filter* function in package poppr we collapsed chlorotypes having only one difference, which resulted in eight chlorotypes (S5B Fig). This allowed suggesting an American species as female parent for AES6 and *V*. *vinifera* for LAB2 (cv. Concord).

### PCA analysis of morphological traits

**Fig 6:**
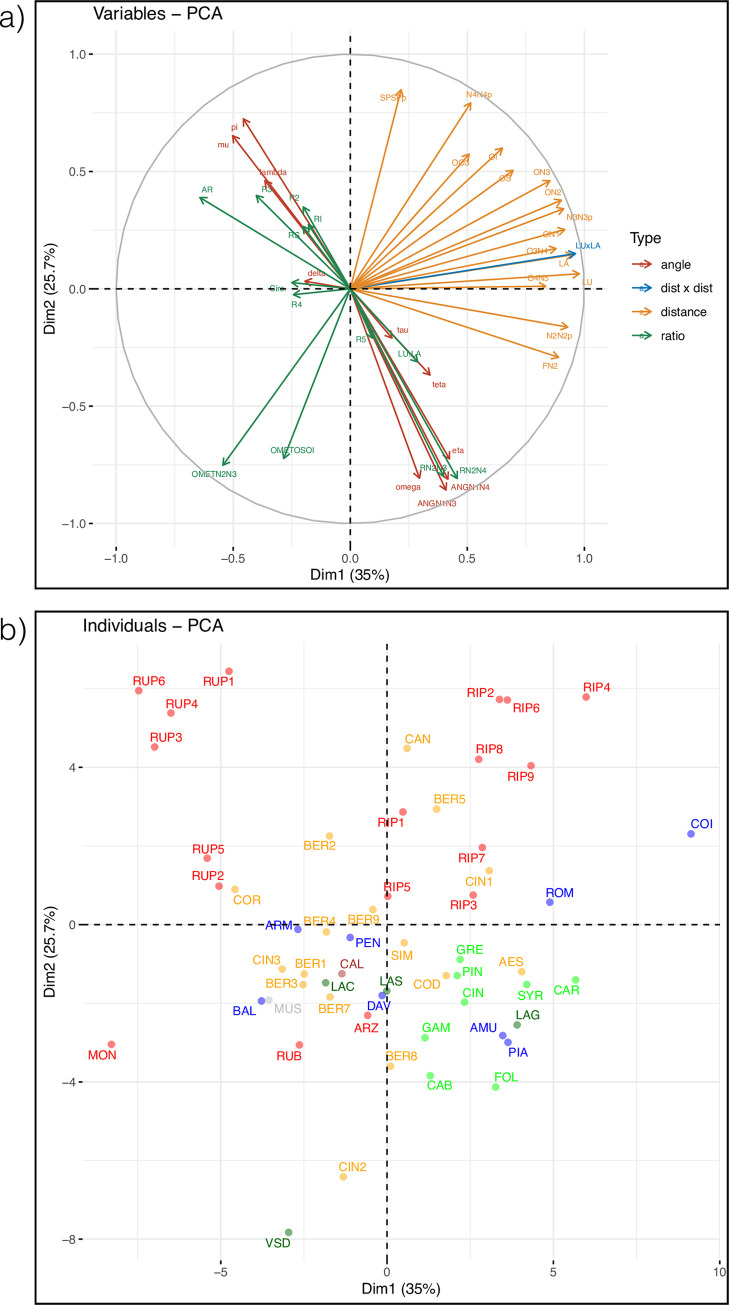
PCA using leaf measures. a) correlation plot for variables classified according to their type (length, angle and ratio, see S4 Table) b) plot for *Vitis* accessions grouped by clades identified using molecular data and colored as in Fig 1. Hybrids were excluded.

In the PCA with the 39 leaf measures (Fig 6), the two first axes explained 60.7% of the variance. PC1 (35% of the variance) reflected the effect of many length variables (Fig 6A) and separated the accessions according to their leaf size (Fig 6B). Species with large leaves such as *V*. *coignetiae* (COI), *V*. *romanetii* (ROM), *V*. *riparia* (RIP) and some cultivars of *V*. *vinifera* (e.g. Carignan, CAR) were distinguished from species with small leaves such as *V*. *monticola* (MON) and *V*. *rupestris* (RUP). The variables most contributing to PC2 (25.7% of the variance) were angles and calculated ratio; both separated the accessions according to leaf form. For instance, the distance SPSP’ and the angle pi both allowed distinguishing species with a large sinus opening: *V*. *rupestris* (RUP) and *V*. *riparia* (RIP). The angles between the three main veins N1,N2 and N3 and especially their sums grouped accessions having lobes (mostly the sampled *vinifera* accessions). Finally, some calculated variables such as the ratio OMETN2N3 (omega+eta/lengthN2+lengthN3) also appeared useful to define the leaf form and was only correlated with OMETOSOI (omega+eta/OS+OI) which depended on sinuses depth. In addition to these general trends, a rather large variation was noticeable within species having several accessions. Another PCA was performed, keeping only the angles and calculated variables (except leaf area) to better interpret the differences in leaf form among species (S6 Fig). *V*. *rupestris* was easily distinguished on PC1 (38.1% of the variance) from all other species due to its reniform leaves (high leaf width relative to length AR, high values of mu and lambda) with large open petiolar sinus (high values of pi). Other species were separated along PC2 (23.0% of the variance) according to variables measuring lobbing and serration such as Circularity (Circ.) and sinus depth (RS, RI, OMETOSOI). *V*. *vinifera* and some Asian species such as *V*. *piasezki* and *V*. *amurensis*) were lobed and serrated in contrast to other Asian species such as *V*. *armata*, *V*. *pentagona*, *V*. *balanseana*, whose had entire cordiform leaves.

**Fig 7:**
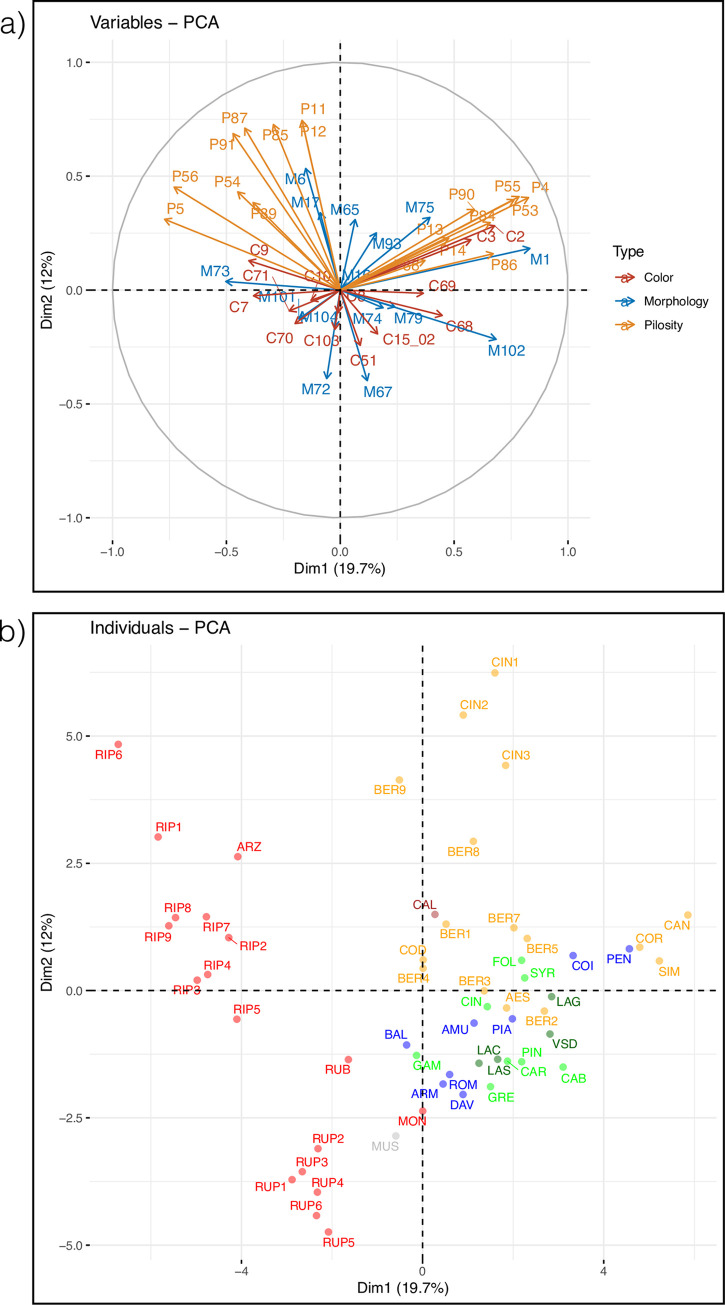
PCA using OIV codes. a) correlation plot for OIV codes classified according to their type (M: Morphology, C: Color, P: Pilosity, see S5 Table) b) plot for *Vitis* accessions grouped by clades identified using molecular data and colored as in Fig 1. Hybrids were excluded.

**Fig 8:**
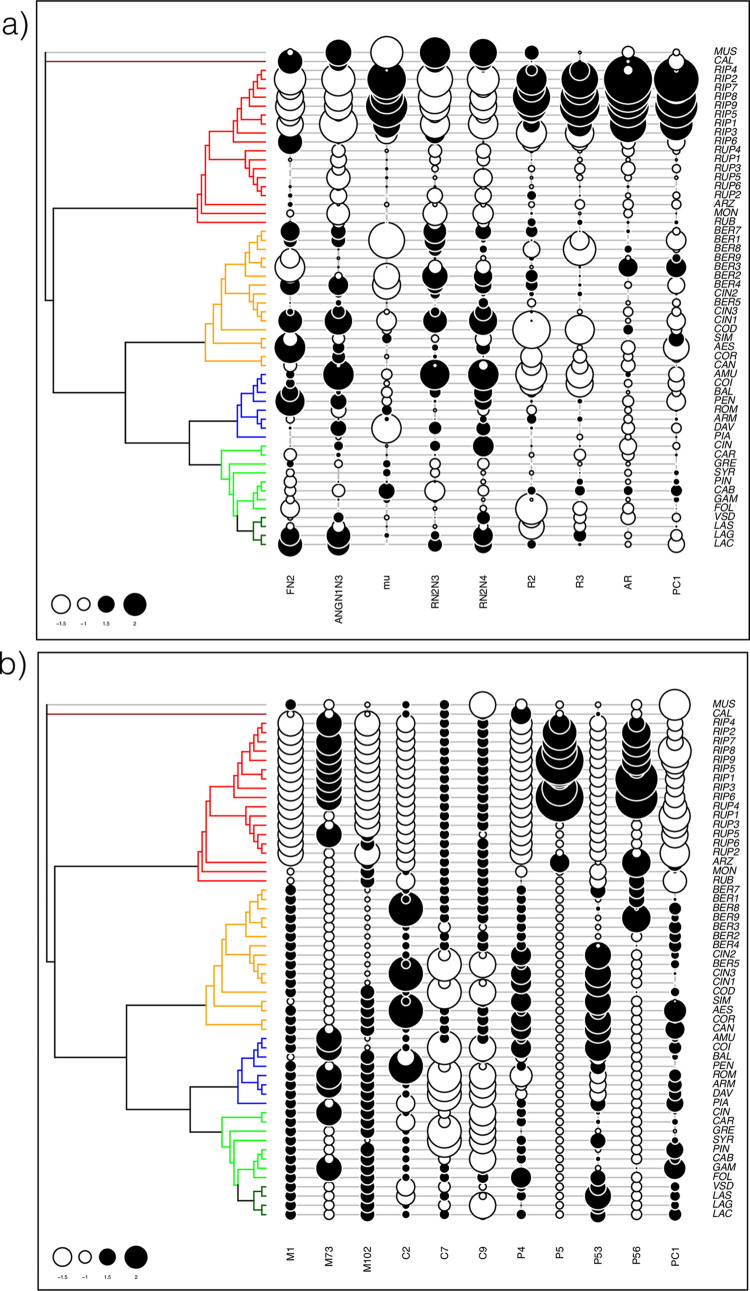
Phylogenetic tree along with the phylogenetically structured traits. a) eight leaf measurements, b) ten OIV codes. The first global principal component of the pPCA (PC1) is shown. Positive and negative values are represented using black and white symbols, respectively, with a size proportional to the absolute value. In the phylogenetic tree clades are colored as in Fig 1. Hybrids were excluded.

For the traits coded using OIV scales, the two first PCA axes explained 31.7% of the variance (19.7% for PC1 and 12% for PC2) and the contribution of most variables was weak (Fig 7A). However, the different genetic clades and also species within clades were clearly distinguished (Fig 7B). PC1 separated species from the NA2 clade whereas PC2 tended to separate NA1 to EU and EA clades. Opening of the shoot tip (M1 = OIV001), surface relief (M102 = OIV102) and several OIV descriptors measuring organ pilosity were the most contributing traits to PC1, the most villous species of clade NA1 (*V*. *candicans*, *V*. *coriacea*, *V*. *simpsonii*) and the glabrous species of clade NA2 such as *V*. *riparia* and *V arizonica* were at opposite sides of PC1. In contrast to the previous analyses with leaf measures, accessions from the same species or species subgroup within clades showed lower variation and clustered in the PCA plot (Fig 8B). This was the case, for instance, for *V*. *cinerea* var. *cinerea* accessions or for the group of Asian species having spines on internodes (*V*. *armata*, *V*. *romanetii*, *V*. *davidii*) although this latter characteristic was not encoded by OIV.

### Searching for a phylogenetic signal

We found 21 and 4 leaf measures with significant autocorrelation at p<0.01 and 0.05, respectively, on a total of 39 measures (listed in S4 Table). The results of pPCA indicated that the variation in these traits corresponded to a global signal (S7A Fig) and the main component (PC1) isolated *V*. *riparia* from the other species (S8A Fig). We then selected the measures having the highest loadings in the analysis (quantile (a,0.1) | a>quantile (a,0.9)) and drew a plot (Fig 8A) using only the eight selected variables, some of which being correlated (S9A Fig). These traits were characteristics of leaf form: distance between F and N2 extremity (FN2), angle between veins 1 and 3 (ANGN1N3: omega + eta), ratios of leaf width measured at vein extremities (RN2N3, RN2N4), angle mu and ratio between major and minor axis (AR).

We found 30 and 5 OIV descriptors with significant autocorrelation at p<0.01 and 0.05, respectively, on a total of 46 descriptors (S6 Table). The results of pPCA indicated that the variation corresponded to a global signal (S7B Fig) and the main component (PC1) isolated the whole NA2 clade + *M*. *rotundifolia* from all the other species (S8b Fig). We then selected the variables as above and drew another plot using the 11 variables having the highest loadings (Fig 7B), some of which being correlated (S9B Fig). The most structured traits corresponded to all three categories: morphology (M1 = OIV001: opening of the shoot tip; M102 = OIV102: surface relief, M73 = OIV073: undulation between veins), coloring (C2 = OIV002: distribution of anthocyanin coloration on prostrate hairs of the shoot; C7 = OIV007: color of the dorsal side of internodes; C9 = OIV009: color of the dorsal side of nodes) and villosity (P4 = OIV004: density of prostrate hairs on the shoot tip; P5 = OIV005: density of erect hairs on the shoot tip, P53 = OIV053: density of prostrate hairs between main veins on lower side of blade (4th leaf); P56 = OIV56: density of erect hairs on main veins on lower side of blade (4th leaf)).

## Discussion

### The *Vitis* genus comprises several clades

In the last decade, numerous efforts have been made to obtain a comprehensive description of species relationships within the *Vitis* genus. Phylogenetic studies have concerned both the chloroplast [17–19, 26, 28] and nuclear [18–25, 27, 47] genome using different sets of markers and accessions. All these works agreed with the sister position of subg. *Muscadinia* relative to subg. *Vitis* and most of them concluded that subg. *Vitis* may be divided in several clades corresponding to the continental origin of the species. However, incongruent results have been frequently observed for the placement of either clades in subg. *Vitis* or species within clades. These discrepancies have been attributed to non-exclusive reasons such as recent divergence, natural hybridization or false identification of accessions. Another additional explanation might be the type of markers or the manner they were obtained. For instance, previously widely used markers such as Simple Sequence Repeats (SSR) were prone to homoplasy [68]. More recent technologies also have some biases, due to the procedures used to select SNPs in the case of SNP arrays [69, 70] and to the part of reduced genome examined by genotyping-by-sequencing [71]. Using a very large number of markers issued from whole-genome sequencing did not always guaranty a more precise description of the species relationships. For instance in grape, *V*. *cinerea* and *V*. *aestivalis* were not recovered as monophyletic in a whole-plastome tree [26]. As another example, the relationships among American species were poorly described within the ML tree obtained after the complete nuclear genome sequencing of 472 *Vitis* accessions [27, S3 Fig]. Quality of sequences, parametrization in the bioinformatic process, use of a reference sequence from *V*. *vinifera* and high heterozygosity in nuclear data could introduce additional difficulties. Furthermore, to assess species relationships even in a recent divergent genus, using several thousand or millions of markers do not seem necessary in contrast for example to genetic association studies [72].

In this work, we first used Sanger sequencing to discover polymorphims in a limited number of loci. ML tree based on these polymorphims confirmed i) the outgroup position of *M*. *rotundifolia*, ii) the peculiar position of *V*. *californica*, iii) the existence of two other clades in North America, iv) a clade comprising all Asian species and v) the derived position of *V*. *vinifera* in Eurasia. In addition, accessions of the same species generally clustered together. We then used an SNP array comprising around thirty times more SNPs than the Sanger data and obtained a similar phylogeny with a higher support for the main nodes. We demonstrated that the ascertainment bias due to the discovery panel had large effects on branch lengths with a lower discrimination of species in clades not included in the panel (e.g. North American clade 2). Moreover, some species (e.g. *V*. *rubra*, *V*. *monticola*, *V*. *candicans*) having a sister position in clades according to the ML tree (Sanger data) exhibited an ambiguous position in the distance tree (SNP array data). This could be due to the absence in the array of the polymorphisms specific of these more distant species. We therefore considered that the ML tree obtained with sequence data better reflected the species relationships.

### Hybridization in *Vitis* genus

Natural hybrids in the genus *Vitis* have long been recognized based on morphological and agronomic traits, especially in North America [73]. For instance, Foex [74] suggested, as early as 1882, that *V*. × *champinii* was a hybrid combining the vigor of *V*. *candicans* and the rooting ability and hardiness of *V*. *rupestris*. Several parentages proposed in the past have indeed been supported by recent molecular data: Zecca et al [30] using data from the 9K SNP array [75] obtained evidence that *V*. × *champinii* and *V*. × *doaniana* resulted from natural crosses between *V*. *candicans* and, respectively, *V*. *rupestris* and *V*. *acerifolia*. In the present work, we reached a similar conclusion based on haplotype networks of a few nuclear loci for *V*. *× champinii*, whereas fo *V*. *× doaniana* the second parent might be *V*. *riparia* or the close species *V*. *longii* (syn. *V*. *acerifolia)*. Moreover, cpDNA polymorphisms allowed us to infer the direction of crosses. Their hybrid origin might not be deduced form their position in the ML-tree since they clustered with one of their parent (*V*. *candicans*) within the same clade (NA1). By contrast, artificial hybrids between *V*. *vinifera* and *V*. *labrusca* (also called *V*. *labruscana*) occupied intermediate positions in the ML and distance trees. Our cpDNA data showed that the female parent is *V*. *labrusca* for cv. Isabelle and that the chlorotype of cv. Concord originated from *V*. *vinifera*. This latter result confirms the recent study of Wen et al. [76] who also pointed out a probable backcross with *V*. *labrusca* as already suggested by Huber et al. [77].

The previous examples indicated that the position of hybrids in phylogenetic trees is not predictable and depends on which parent the hybrid shares the most derived characters with [78]. We therefore used information not only from the trees but also from haplotype networks and the heterozygosity values to define the most probable hybrids in our sample. A total of eight accessions were confirmed or identified as hybrids and have been eliminated from downstream analyses. We considered this step as essential to provide an accurate view of the relationships between the pure species. However, we acknowledge the fact that possible hybrids within clades or complex interspecific hybrids may have escaped from our scrutiny at this stage.

Zecca et al [30] detected a very interesting gene flow between the *M*. *rotundifolia* outgroup and two species from clade NA1 *V*. *candicans* (syn. *V*. *mustagensis*) and *V*. *coriacea* (syn. *V*. *shuttleworthii*), which are both currently sympatric with *M*. *rotundifolia*. It had been also previously documented that this species hybridized with several American *Vitis* species and more readily when used as a male than a female parent [79]. The existence of introgression between the two subgenera in North America would bring the outgroup closer to American species in phylogenetic trees. This could have provided a false support for the American origin of subg. *Vitis* suggested in several studies [22, 23, 25].

### Species relationships within clades

For *V*. *vinifera* (clade EU), the accessions included in our work only covered a little part of the species diversity. The cultivars of subsp. *vinifera* analyzed represented mostly the diversity group from Western Europe and have a wine usage [51, 80]. The studied wild accessions of subsp. *sylvestris* also originated from the same region except the Tunisian accession (LAS). In Western Europe, the differentiation of the two subspecies is probably limited due to the existence of a gene flow [81]. However, the two subspecies were clearly separated using the sequence data without the hybrids (Fig 3). The SNP array data without hybrids also separated the two subspecies, although the Tunisian accession clustered or not with the cultivated accessions depending on the set and number of SNPs used (Fig 5).

The East Asian clade is sister to the European clade. The sequence data allowed to cluster some Asian species: i) *V*. *amurensis*, *V*. *coignetiae* and *V*. *thunbergii* that constitute a northern clade, ii) *V*. *flexuosa* and *V*. *balanseana* representing the *V*. *bryoniifolia* clade recently studied by Ma et al [41] and iii) *V*. *armata*, *V*. *romanetii* and *V*. *davidii* included by Galet [12] in the *Spinosae* serie. Several discrepancies were observed between the sequence and array data. For instance, *V*. *amurensis* was found sister to other Asian taxa in the distance tree but not in the ML tree. Moreover, in the distance tree *V*. *armata* did not cluster with the two other species of the *Spinosae* serie, which was identified using sequence data. These differences could be due to the lack of Asian taxa within the discovery panel for the SNP array.

Our results for American species are largely congruent with those of Klein et al [24] who also described two distinct groups: Clade I and II corresponding to our clades NA2 and NA1, respectively. These authors discussed in details the characters of each of the clades in relation with previous classification, morphological characters and phylogeography. NA1 clade includes species from eastern regions like *V*. *aestivalis*, *V*. *cinerea*, *V*. *cordifolia* (syn. *V*. *vulpina*), V. *labrusca*, V. *coriacea* (syn. V. *shuttleworthi*), V. *simpsonii* and V. *candicans* (syn. *V*. *mustangensis*). Both sequence and array data identified a *V*. *cinerea* clade including the two varieties var. *cinerea* and var. *berlandieri (*syn. var. *helleri*). These varieties were clearly distinguished especially in the distance tree with a strong support (Fig 7D). Gene flow between them is probably limited due to the strict adaptation of var. *berlandieri* to dry climate and lime soils [82]. Some authors proposed to classify var. *berlandieri* and var. *cinerea* in two different species based on morphological differences [83]. However, a recent population analysis showed that the two varieties are in the species boundaries and that var. *cinerea* probably contains several genetic groups [84]. Interestingly, our present study identified *V*. *cordifolia* as sister to *V*. *cinerea*.

Further work is required to better understand the relationships among *V*. *aestivalis*, *V*. *simpsonii*, *V*. *coriacea* and *V*. *candicans*. These species share similar climatic niches in the southeastern United States [85] where some morphological similarities and possible hybridization caused taxonomic ambiguities [86]. For instance, our *V*. *coriacea* accession exhibited a high heterozygosity and shared many polymorphisms with *V*. *simpsonii* and *V*. *aestivalis*. Furthermore, the position of the three species in our phylogenetic trees changed according to the data set. Sequence data grouped *V*. *aestivalis* and *V*. *simpsonii* whereas the SNP array data grouped *V*. *simpsonii* and *V*. *coriacea*. *V*. *simpsonii* has sometimes been named *V*. *cinerea var*. *floridana* [87]. By contrast, accessions with this latter name did not group with other *V*. *cinerea* accessions in recent phylogenetic trees based on chloroplast [26] and nuclear [30] polymorphisms. We therefore suggest that *V*. *cinerea* var. *floridana* does not belong to *V*. *cinerea*; it is more probably a pure species or a hybrid close to *V*. *aestivalis* and *V*. *coriacea*.

NA2 clade includes the species: *V*. *arizonica*, *V*. *riparia*, *V*. *longii* (syn. V. *acerifolia*) and *V*. *rupestris*, thus confirming previous results [21, 22, 24]. In addition, we found a very close proximity between *V*. *arizonica* and *V*. *treleasii*. These two species have been assigned to the same series in previous classifications based on morphology [10, 12]. We identified *V*. *monticola* as a sister species in clade NA2 in congruence with previous molecular studies [21, 24]. We obtained conflicting results for *V*. *rubra (*syn. *V*. *palmata)*. Sequence data identify this species as sister to the clade NA2 but SNP array indicated a close genetic relationship with *V*. *candicans* from clade NA1. This species could be a relict and its isolated position in the distance tree may be due to the sharing of ancestral polymorphisms with *M*. *rotundifolia*, which was represented in the discovery panel.

Our results also confirmed the particular phylogenetic position of *V*. *californica*, already pointed in several contributions [e.g. 17–19, 26], this species appearing very different from all other American species. In contrast to Ma et al [25], our nuclear data are congruent with plastome data [e.g. 18, 26]. *V*. *californica* was probably isolated for a long time in northwestern America and then entered in contact and hybridized with *V*. *girdiana* [30, 87] as well as, more recently, with *V*. *vinifera* [87]. *V*. *californica* shared characters with the core subg. *Vitis* from Eurasia: low to high susceptibility to mildew diseases and reddening leaves at fall whereas all other American species display low to high resistance to mildews and have yellowing leaves at fall.

### Adaptation and morphological diversification in subgenus *Vitis*

Subgenus *Vitis* experienced rapid radiation in East Asia [25] and also a rapid and probably more recent radiation in North America [26]. These radiations have been associated with adaptation to diverse ecological conditions implying diversification for morphological characters. Similar adaptation has been observed in both continents, leading to two main growth habits: climbers with large leaves, tall plant stature with strong stems and tendrils (e.g. *V*. *riparia*, *V*. *coignetiae*) and shrubs with tiny leaves, small plant stature, many branches and weak climbing ability (e.g. *V*. *rupestris*, *V*. *pentagona*). A striking example of convergence was the assigment by Galet of *V*. *labrusca* and *V*. *coignetiae* to the Labruscae series, as both species have large and entire leaves but are phylogenetically very distinct (e.g. Fig 3). Recent studies [41, 88] also pointed out the importance of environment-driven evolutionary convergences driven in *Vitis*.

In this context, it is not surprising that our analyses of leaf measurements and OIV traits poorly reflected the species relationships found using DNA polymorphisms. As expected, the size and form of leaves did not allow recovering the differents clades although reniform leaves were only found in clade NA2 (*V*. *rupestris*). PCA analyses of OIV traits tend to isolate NA2 and also to separate NA1 from the EU and EA clades but with large variation within species. We then searched for a phylogenetic signal (i.e. “a tendency for closely related species to possess similar trait values due to descent from a common ancestor” [89, 90]) in our data using a method that took simultaneously into account phylogeny and morphological traits. Several traits were found autocorrelated with the phylogeny and the main result pointed again the NA2clade as the most distinct within the core *Vitis* subg. *Vitis*. Diversification in North America has been therefore very marked with three clades (*V*. *califonica*, NA1 and NA2) probably isolated during a long time. They adapted to different environments, which are very contrasted in Northern America [73, 91]. Assessing the relative roles of common ancestry and adaptation in determining morphological traits appears therefore very challenging at the subgenus level [92].

However, leaf measurements and OIV codes are still very useful for identification of species and varieties within species. Moreover, more precise methods for quantifying leaf shape are now available and have been recently applied to *Vitis* species. These methods performed very well for identification purposes even at the clonal level within *V*. *riparia* and *V*. *rupestris* [38]. The digital reconstruction of grapevine leaves allowed also recently identifying numerous QTL associated with leaf shape [93]. Taking into account the variation along the shoot has recently been found to outperform the classic measures of individual leaves [94].

## Conclusions

In this work, we showed that a relatively low number of SNP markers obtained from sequences of a few nuclear genes are sufficient to recover the main divisions in the *Vitis genus*. Our phylogenetic results were largely similar to other recent phylogenies evidenced for this genus and confirmed the continental distribution of genetically distinct clades. In addition, using haplotype reconstruction of nuclear genes and a few chloroplast markers, we identified hybrids in our sample and demonstrated how they might affect the position of pure species in phylogenetic trees. Despite an evident effect of ascertainment bias, genotyping with an SNP array provided congruent results with the sequence data. There was recently a renewed interest for morphological variation in *Vitis*, not only for classification purposes but also to decipher evolution and adaptation in the genus knowing the genetic relationships between species [25, 29, 41, 80]. Here we contributed to this topic by searching a phylogenetic signal in morphological characters. The signal was generally weak but confirmed that the American clade NA2 is the most genetically divergent in subgenus *Vitis* exhibiting original morphological traits. Further research would aim to identify the ancestral species of clades and to follow their evolutionary history during their geographic extension [85]. For that purpose we suggest to combine different types of data: nuclear and plastid polymorphisms, including indels [42], morphological characters in particular for the leaf form and biochemical characters such as those involved in the flavonoid pathway [95].

## Supporting information

S1 Fig Measurements with Superampelo. (PDF)

S2 Fig Boxplots of mean number of SNPs between haplotypes. *Vitis* accessions were grouped in clades and colored as in Fig 1. Wild and cultivated of *V*. *vinifera* (EU) are grouped. Known hybrids (CHA, DOA1, LAB), colored in pink, showed high values as well as other accessions that were therefore considered as putative hybrids.
(PDF)

S3 Fig Haplotypes networks constructed for eleven loci. a) *GAI*, b) *CHI1*, c) *4275A*, d) *TFL1*, e) *LDOX*, f) *DFR4*, g) *TC1A*, h) *TC1B*, i) *255A*, j)*1526A* and k) *4194A*. Haplotypes are colored as in Fig 1.
(PDF)

S4 Fig Boxplots of mean heterozygosity calculated using the SNP array data. Six hybrids having a *V*. *vinifera* parentage showed very high values. In the Eastern Asian clade, the high value of *V*. *amurensis* questioned its origin. Accessions were grouped in clades colored as in Fig 1. Wild and cultivated of *V*. *vinifera* (EU) are grouped.
(PDF)

S5 Fig Chlorotype sharing among *Vitis* accessions. a) assignment in 19 chlorotypes (MLG = Multi Locus Genotype), b) assignment in eight chlorotypes when one marker difference was allowed. Accessions grouped in clades colored as in Fig 1.
(PDF)

S6 Fig PCA of leaf measurements (angle and ratio). a) correlation plot for variables classified according to their type (angle and ratio) b) plot for *Vitis* accessions, grouped in clades colored as in Fig 1. Hybrids were excluded.
(PDF)

S7 Fig pPCA plots obtained with adephylo. (PDF)

S8 Fig Phylogenetic signals in morphological variables. a) leaf measurements, b) OIV codes.
(PDF)

S9 Fig Correlation plots for variables with significant phylogenetic signal. a) leaf measurements, b) OIV codes.
(PDF)

S1 Table List of accessions studied. (XLSX)

S2 Table List of genes and primers for sequencing. (PDF)

S3 Table Discovery panel and SNPs from the Illumina chip. (PDF)

S4 Table Leaf traits measured and their phylogenetic signal. (PDF)

S5 Table OIV descriptors used. (PDF)

S6 Table Phylogenetic signal in OIV descriptors. (PDF)

S1 Data Archive of molecular and morphological data analyzed. (ZIP)
