# Supplementary figures and images for: Species relationships within the genus Vitis based on molecular and morphological data
Source: PLoS One. 2023 Jul 31;18(7):e0283324. doi: 10.1371/journal.pone.0283324 (PMC10389703; doi:10.1371/journal.pone.0283324)

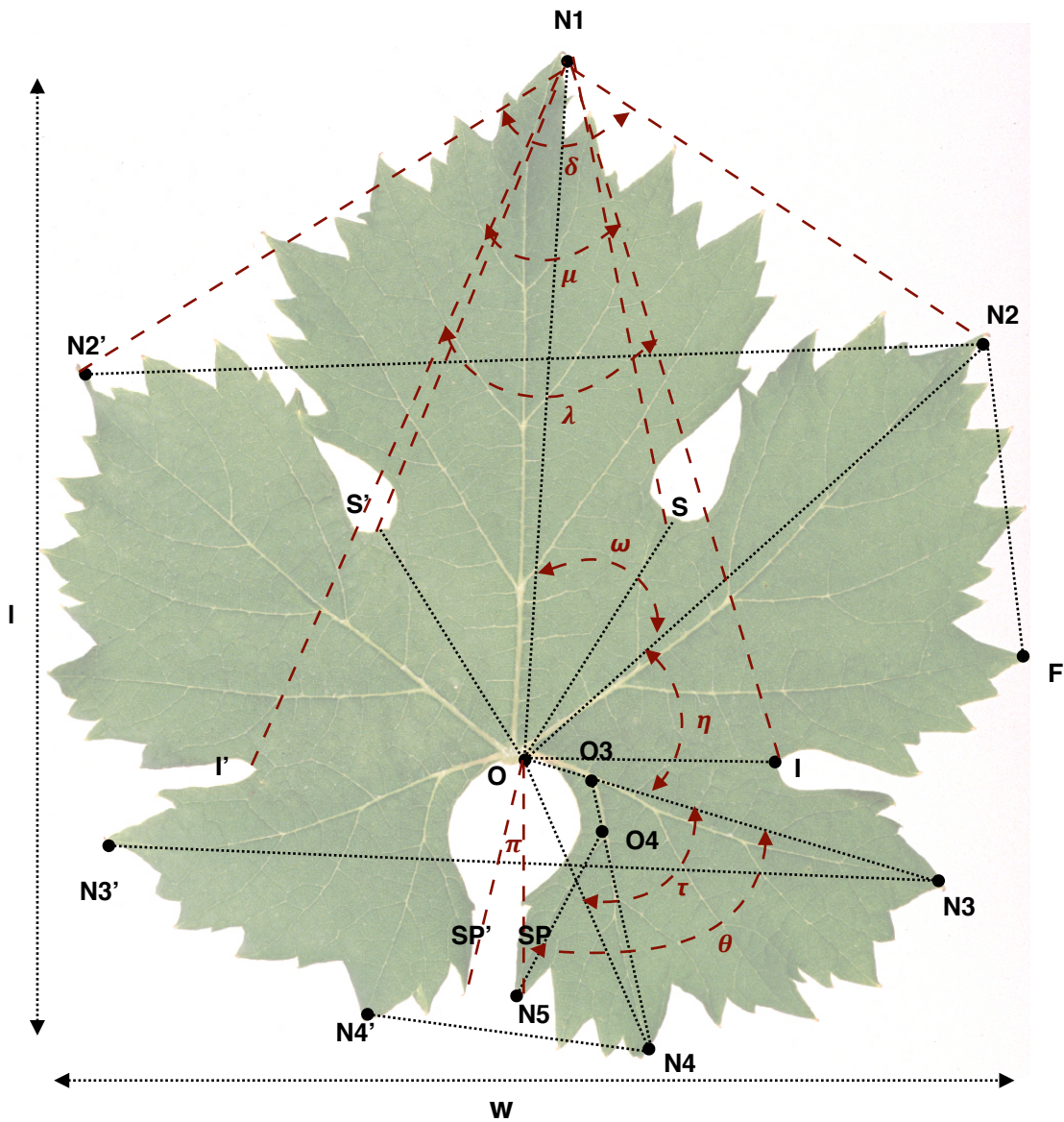

Supplement: S1 Fig — (PDF) [file pone.0283324.s001.pdf]

a) *GAI*

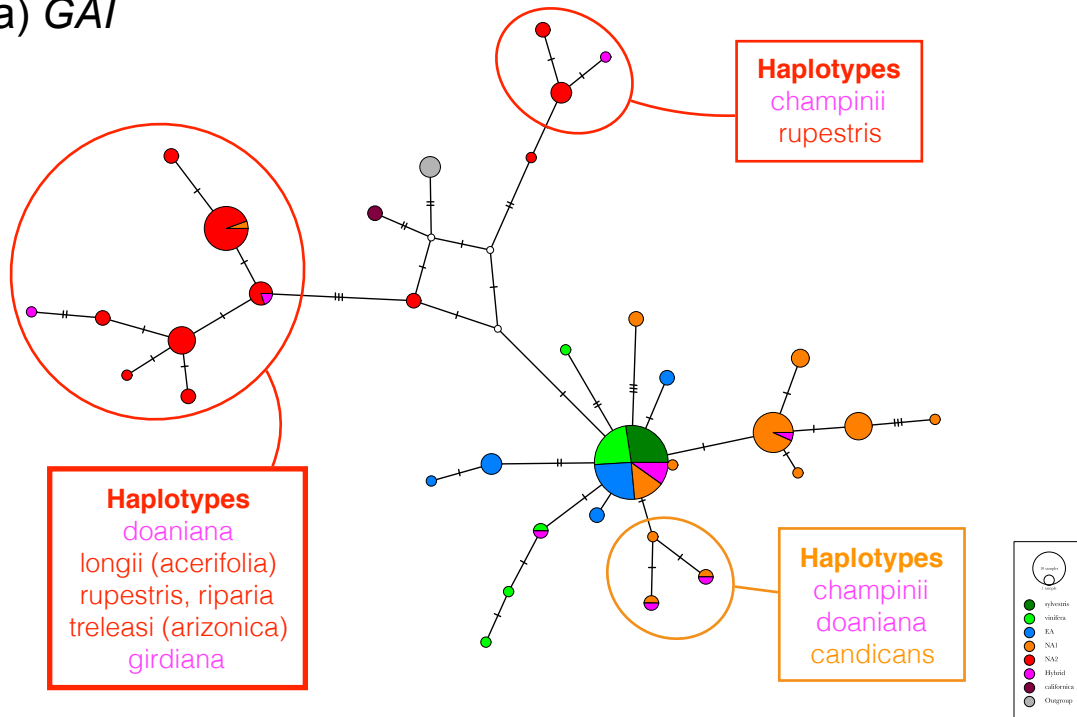

b) *CHI1*

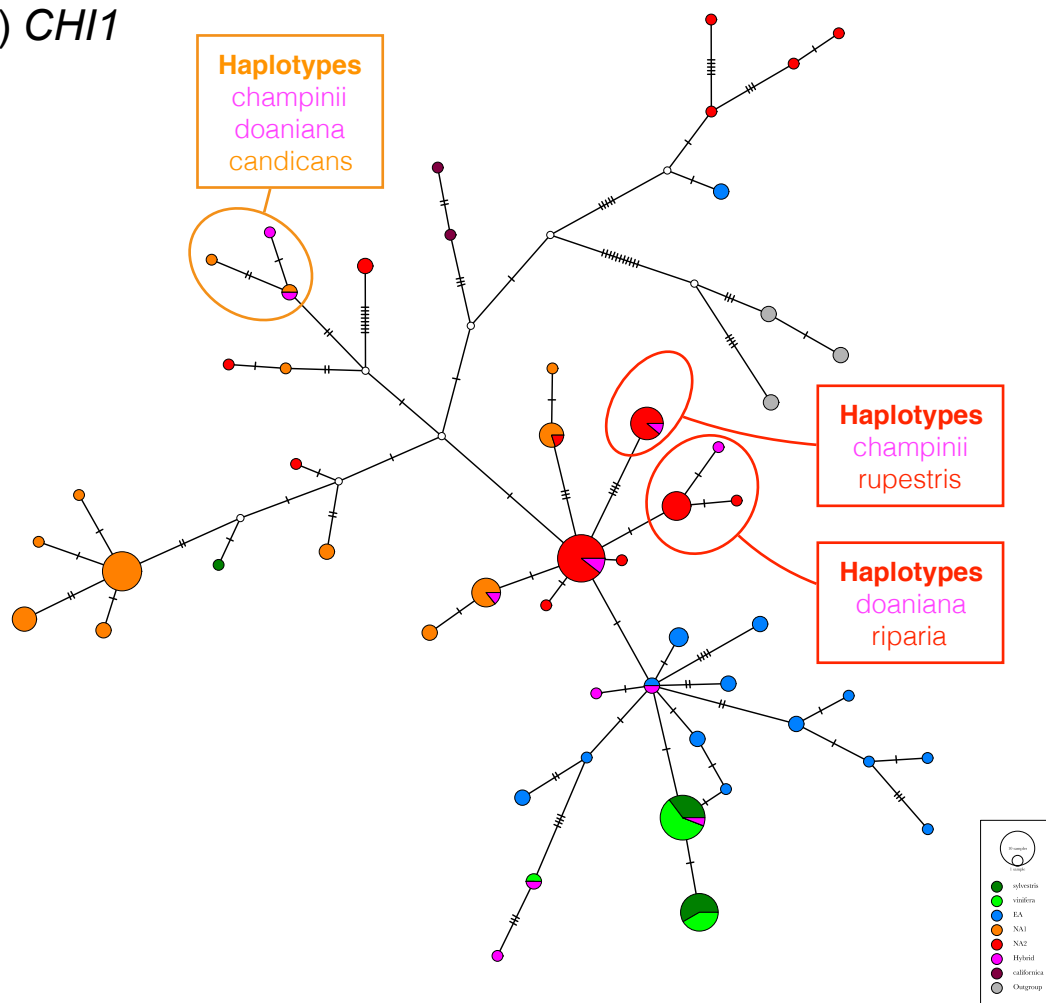

c) 4275A

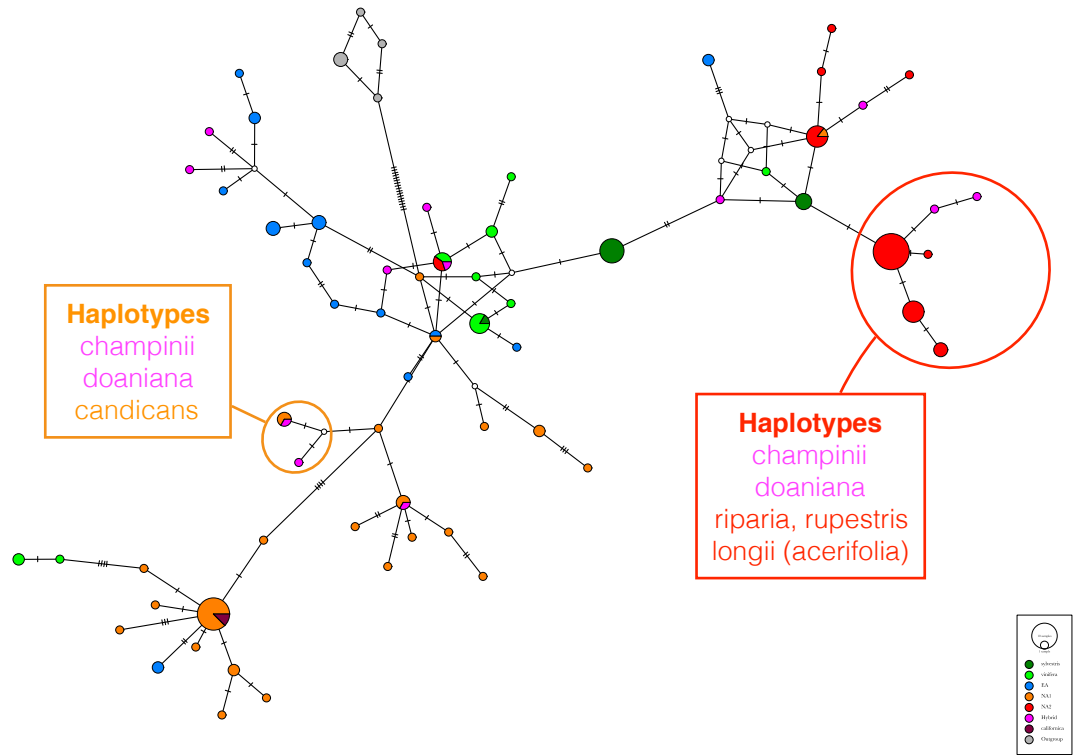

d) *TFL1*

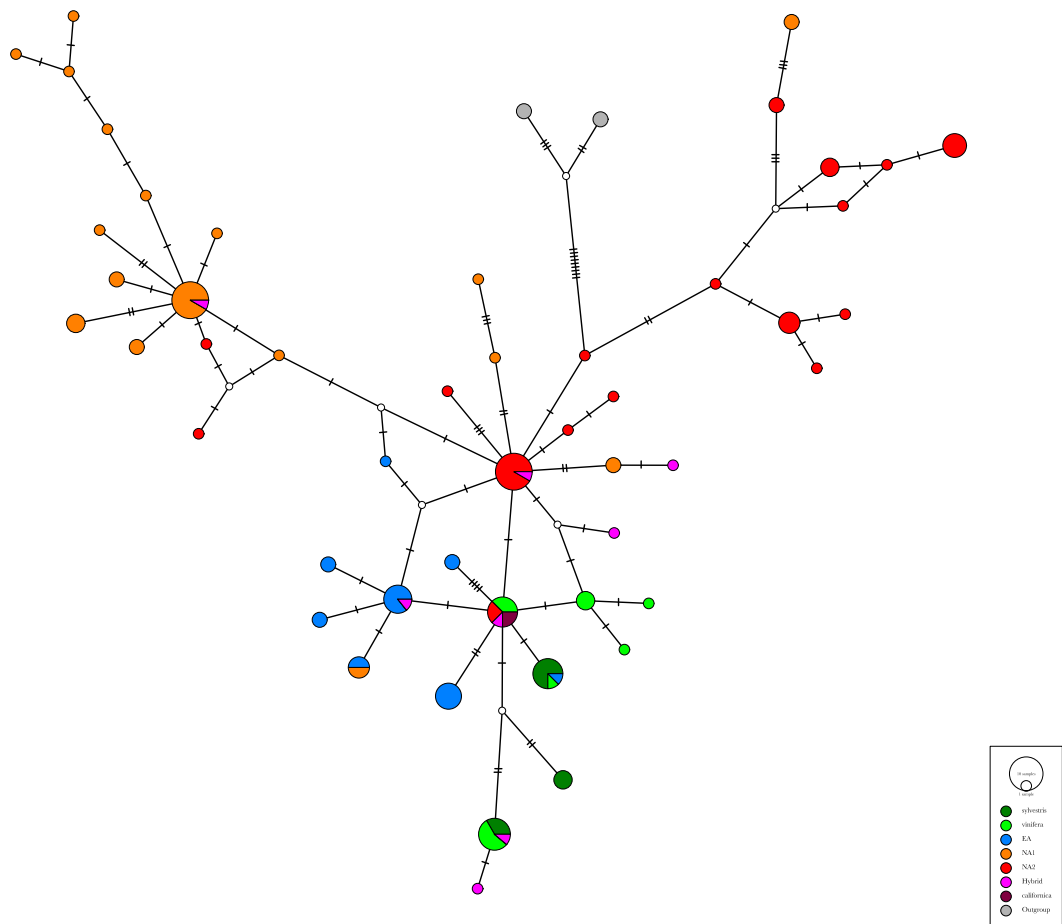

e) *LDOX*

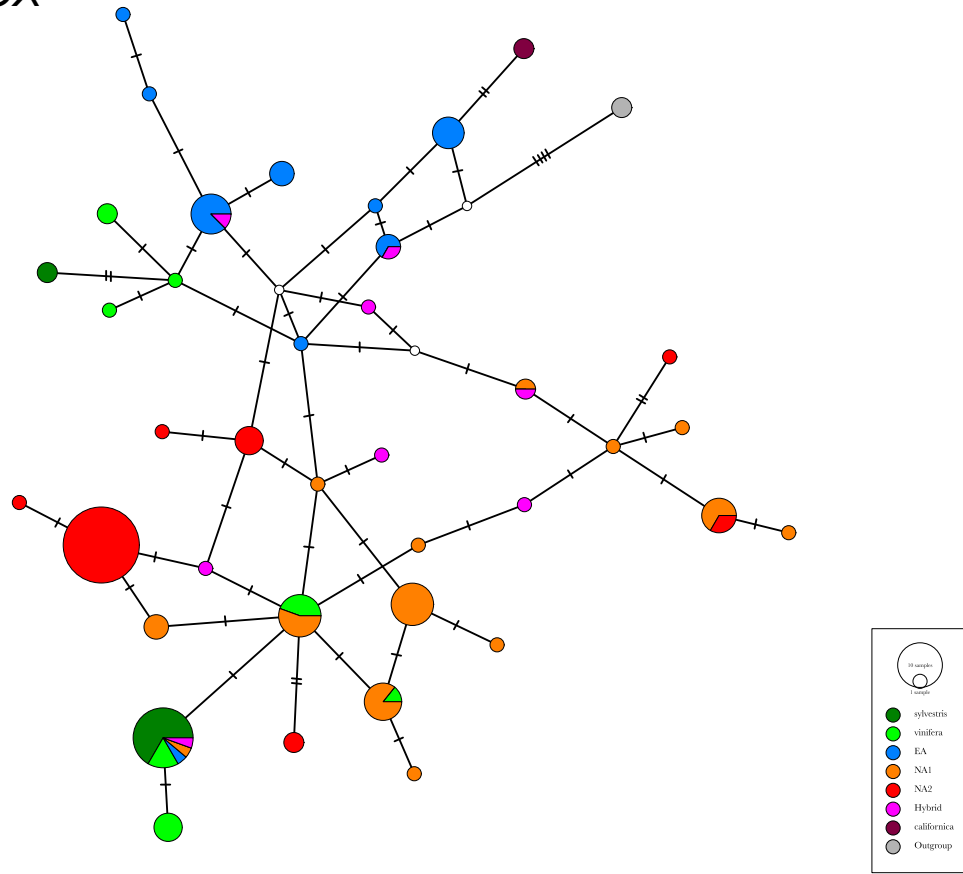

f) *DFR4*

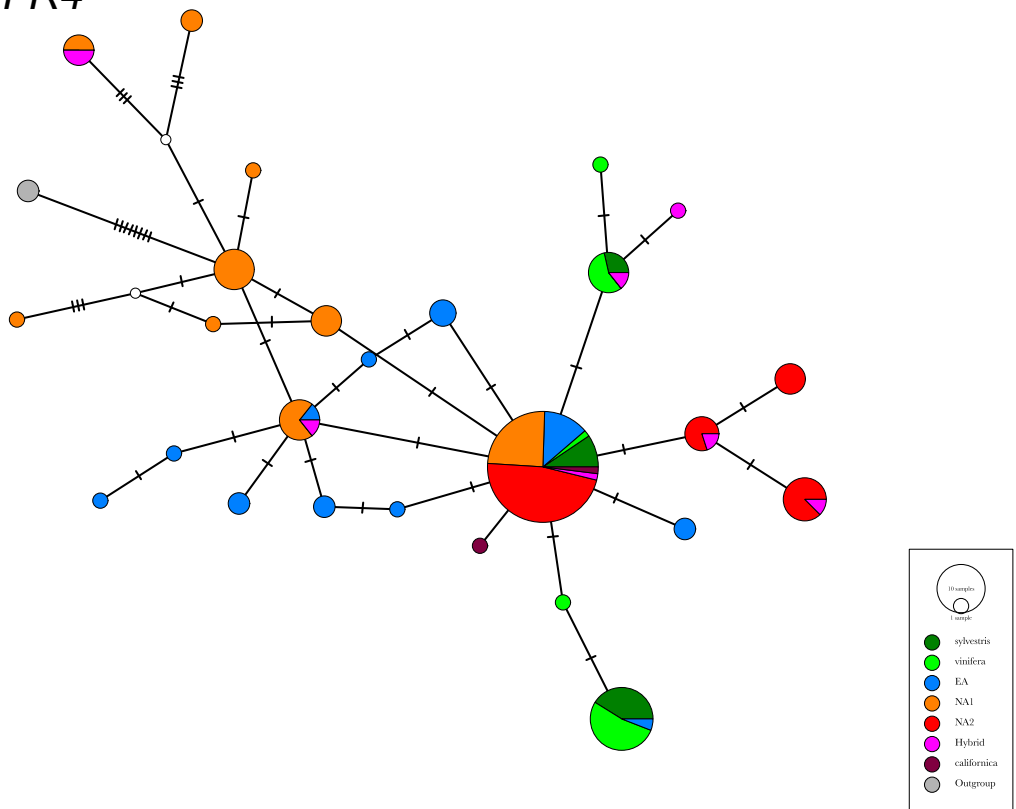

g) *TC1A*

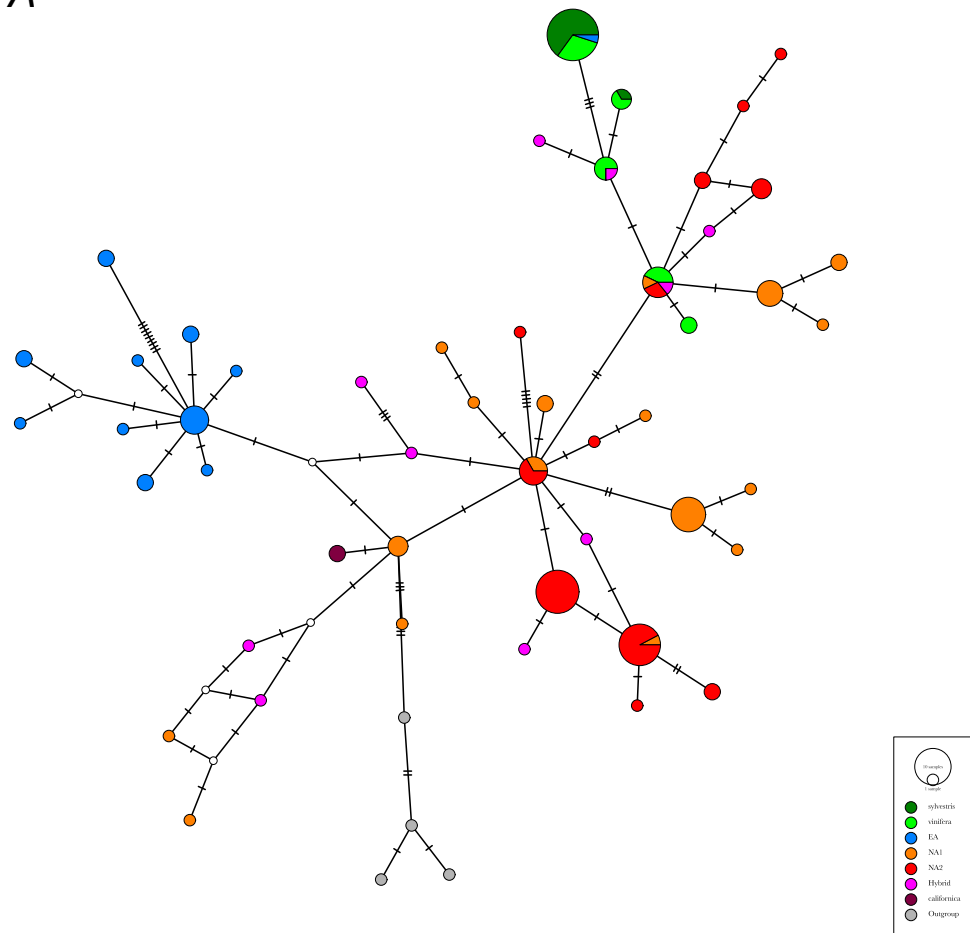

h) *TC1B*

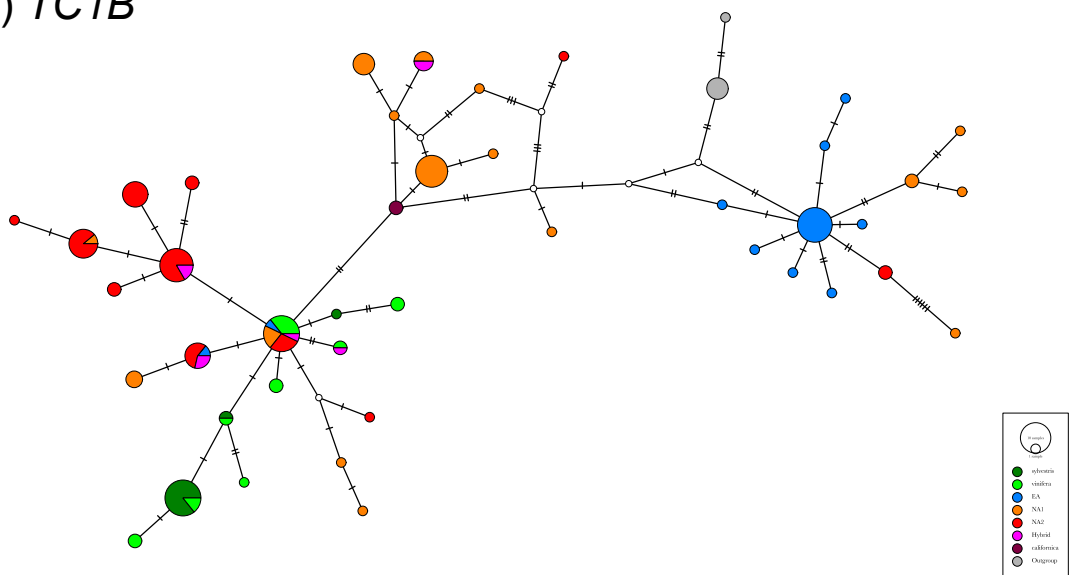

i) 255A

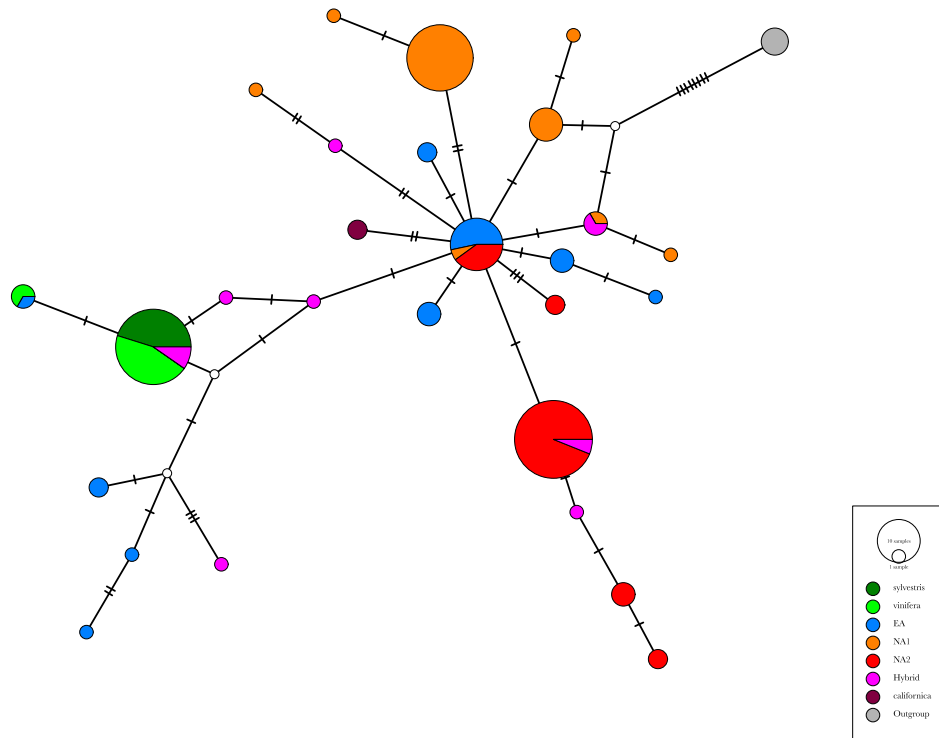

j) 1526A

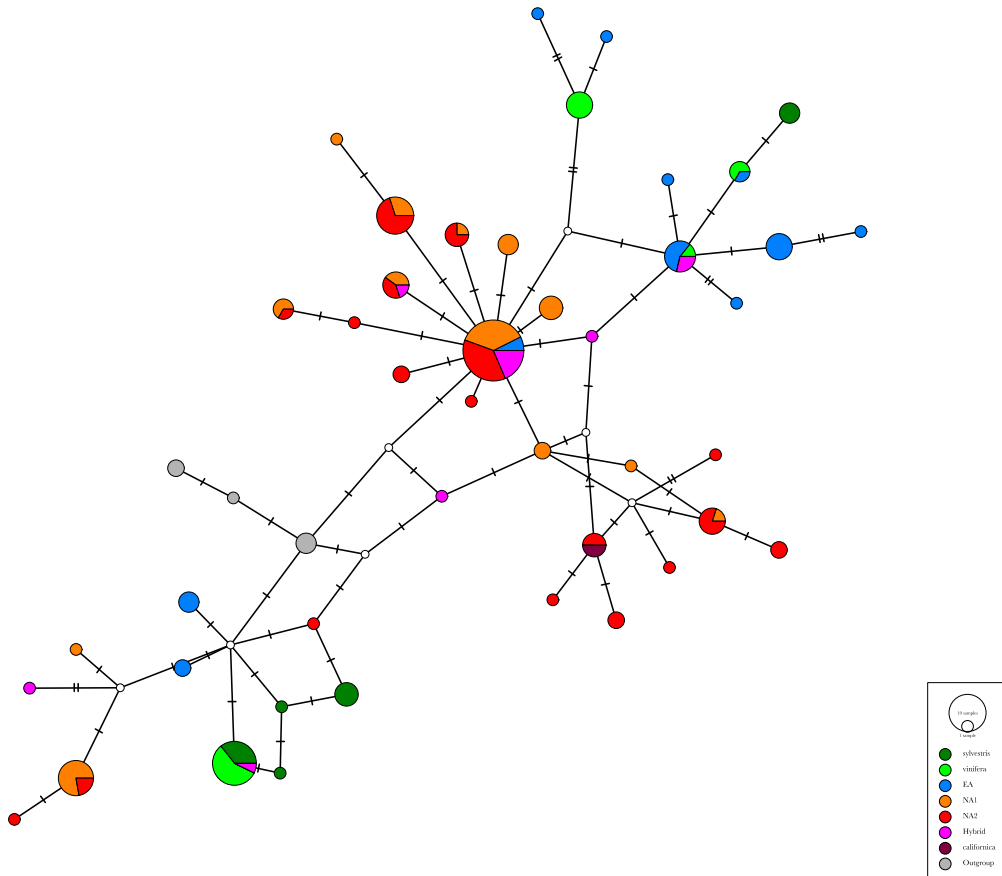

k) 4194A

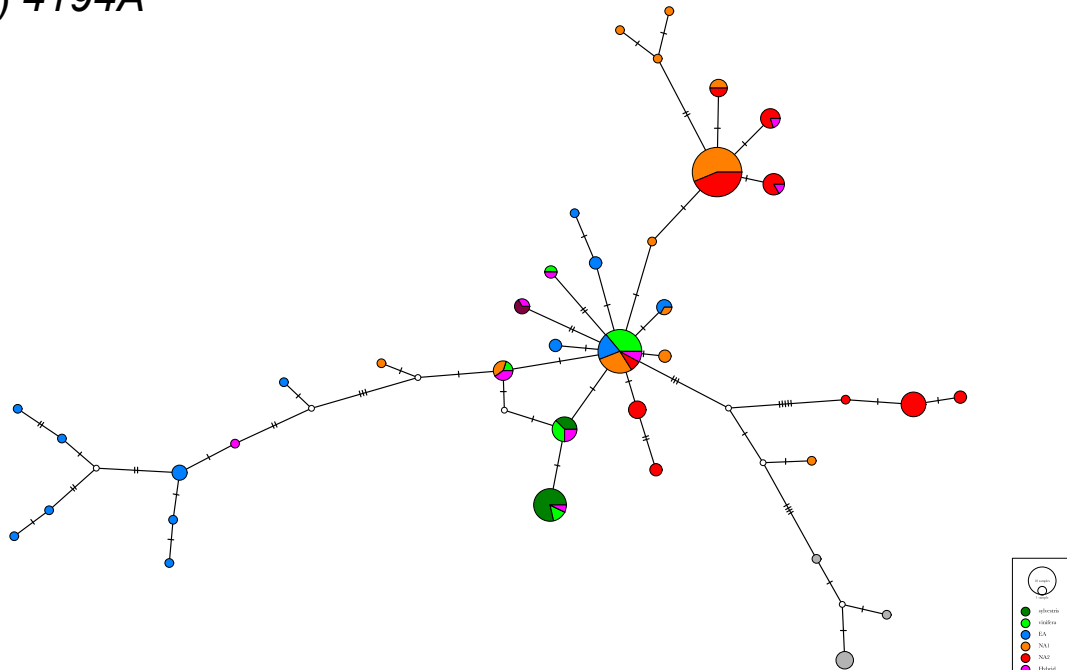

Supplement: S3 Fig — a) GAI, b) CHI1, c) 4275A, d) TFL1, e) LDOX, f) DFR4, g) TC1A, h) TC1B, i) 255A, j)1526A and k) 4194A. Haplotypes are colored as in Fig 1. (PDF) [file pone.0283324.s003.pdf]

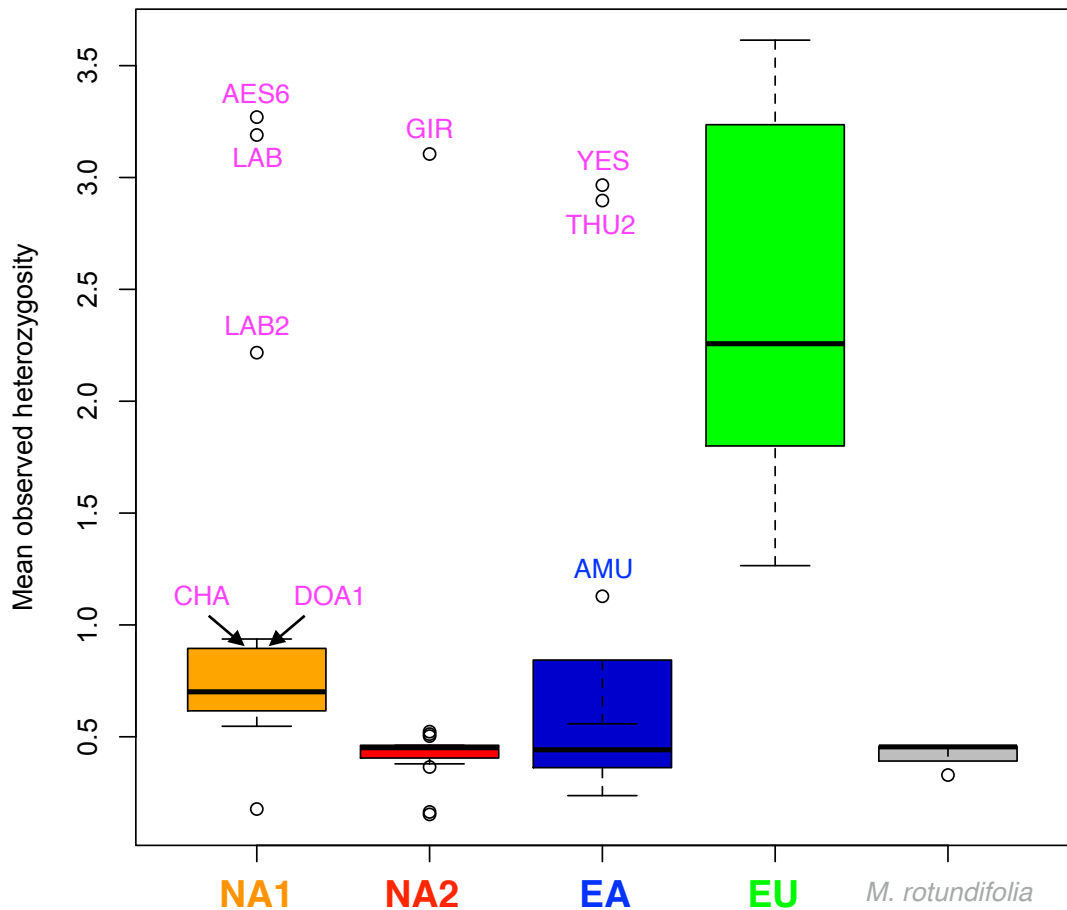

Supplement: S4 Fig — Six hybrids having a V. vinifera parentage showed very high values. In the Eastern Asian clade, the high value of V. amurensis questioned its origin. Accessions were grouped in clades colored as in Fig 1. Wild and cultivated of V. vinifera (EU) are grouped. (PDF) [file pone.0283324.s004.pdf]

a)

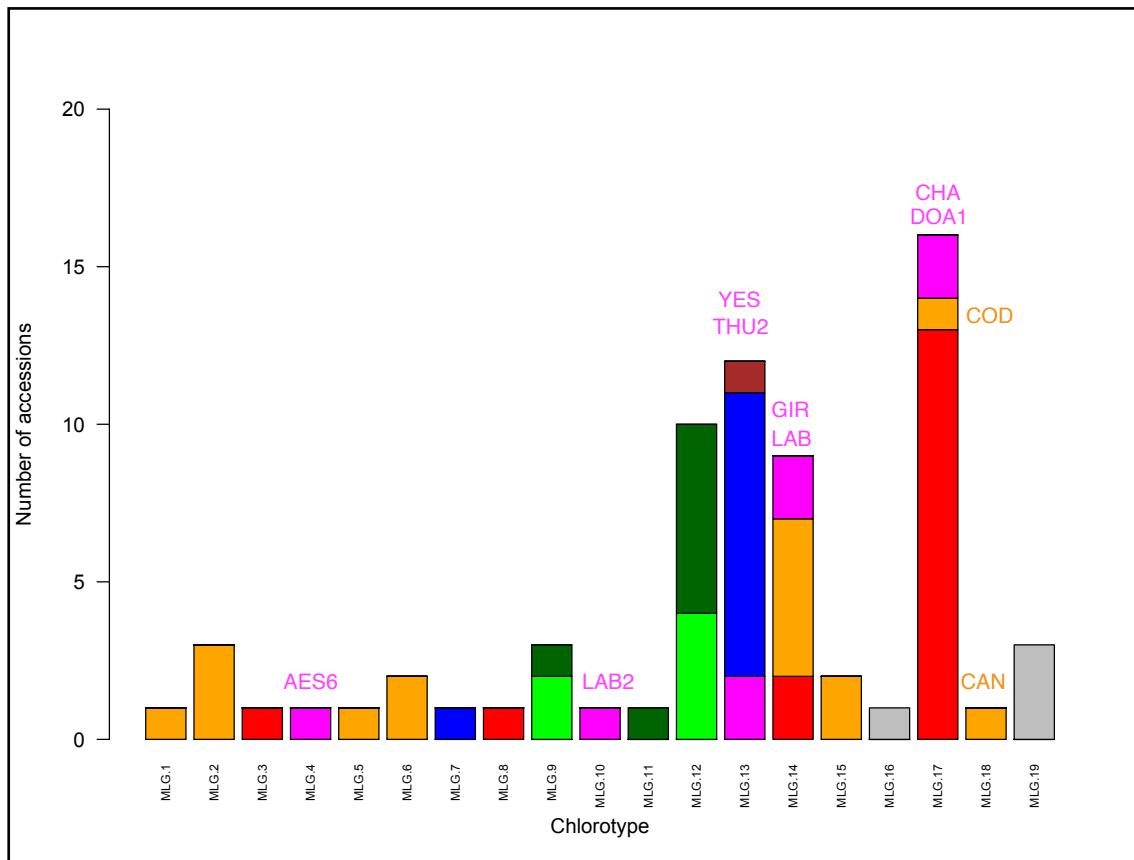

b)

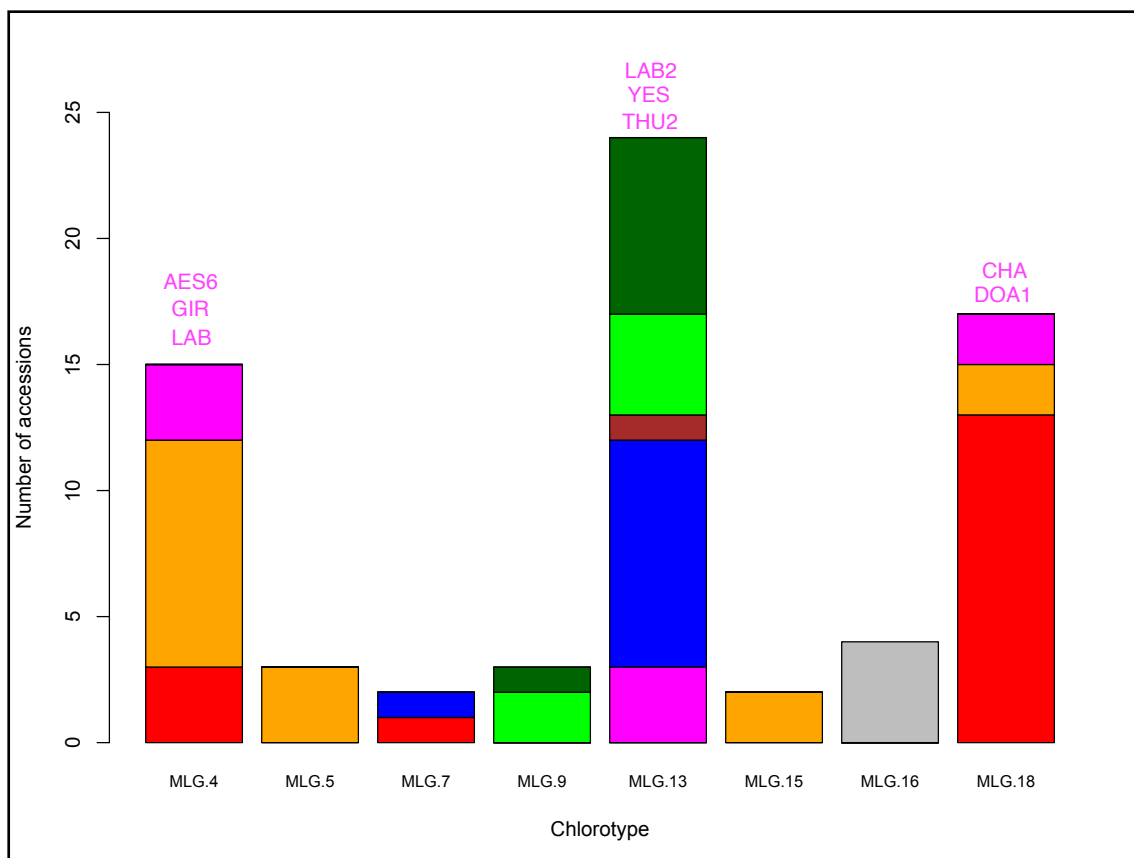

Supplement: S5 Fig — a) assignment in 19 chlorotypes (MLG = Multi Locus Genotype), b) assignment in eight chlorotypes when one marker difference was allowed. Accessions grouped in clades colored as in Fig 1. (PDF) [file pone.0283324.s005.pdf]

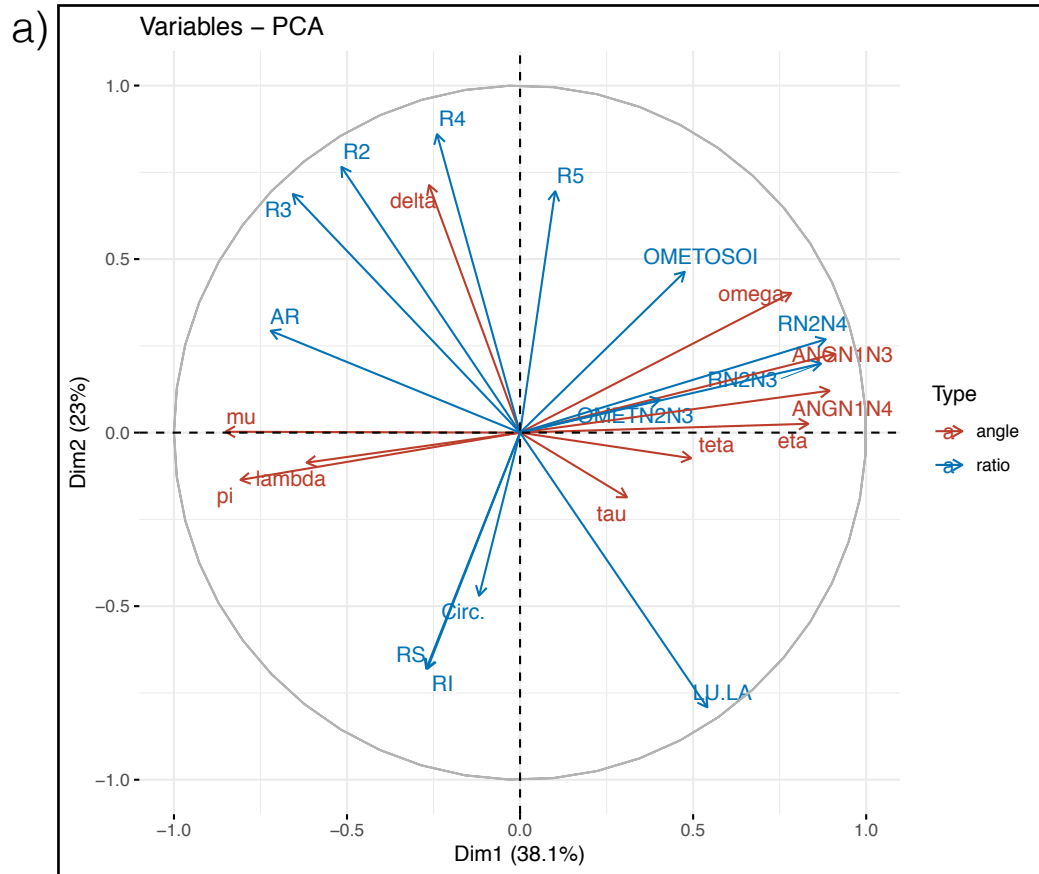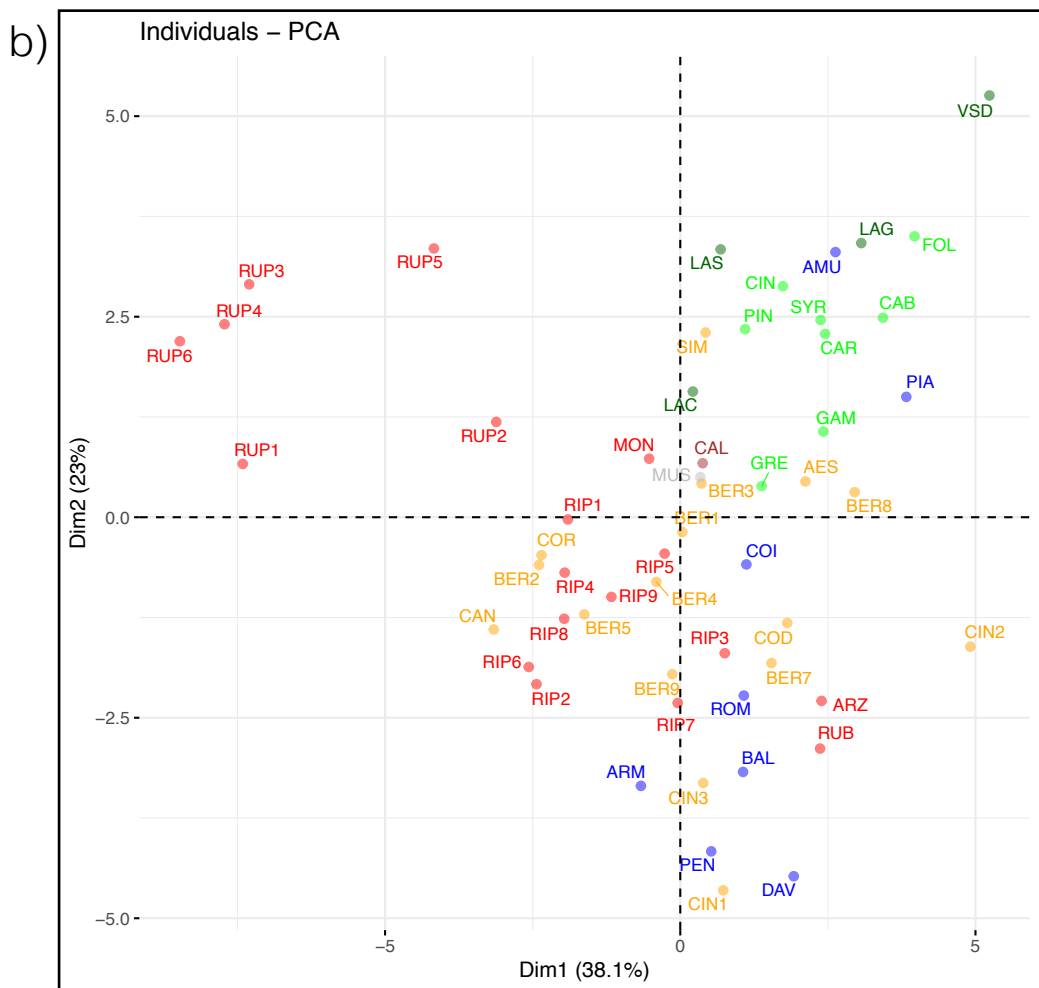

Supplement: S6 Fig — a) correlation plot for variables classified according to their type (angle and ratio) b) plot for Vitis accessions, grouped in clades colored as in Fig 1. Hybrids were excluded. (PDF) [file pone.0283324.s006.pdf]

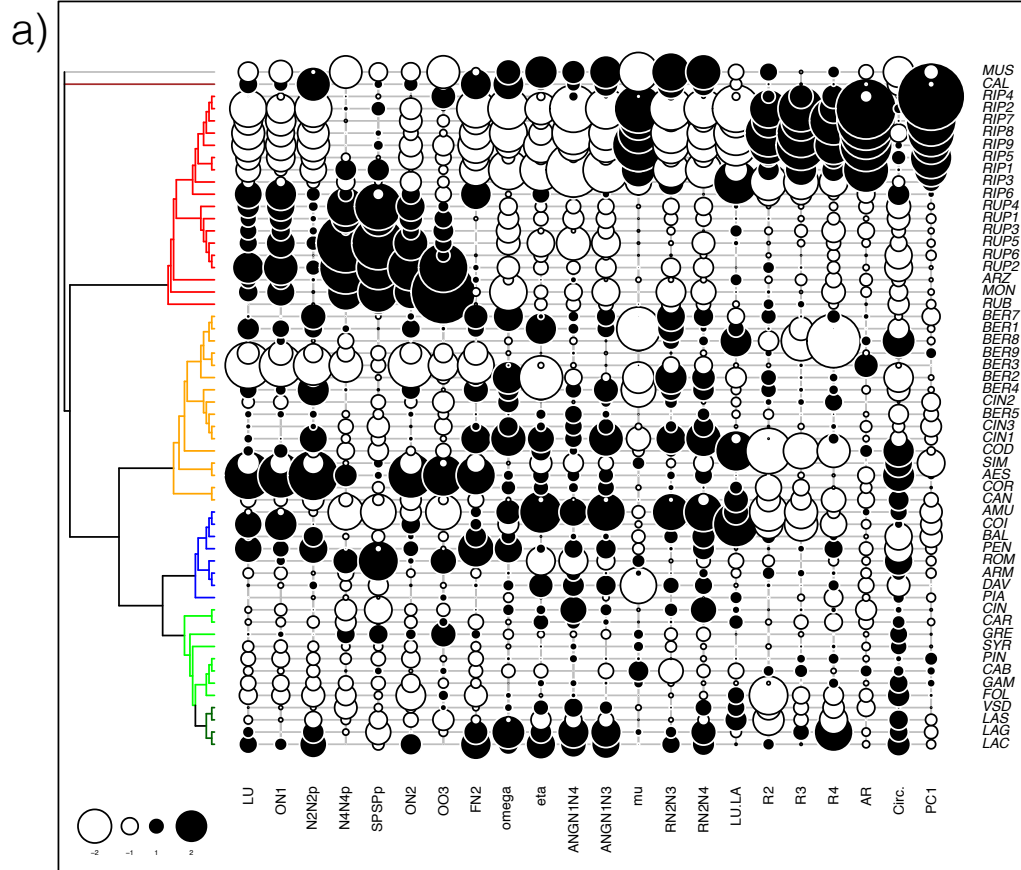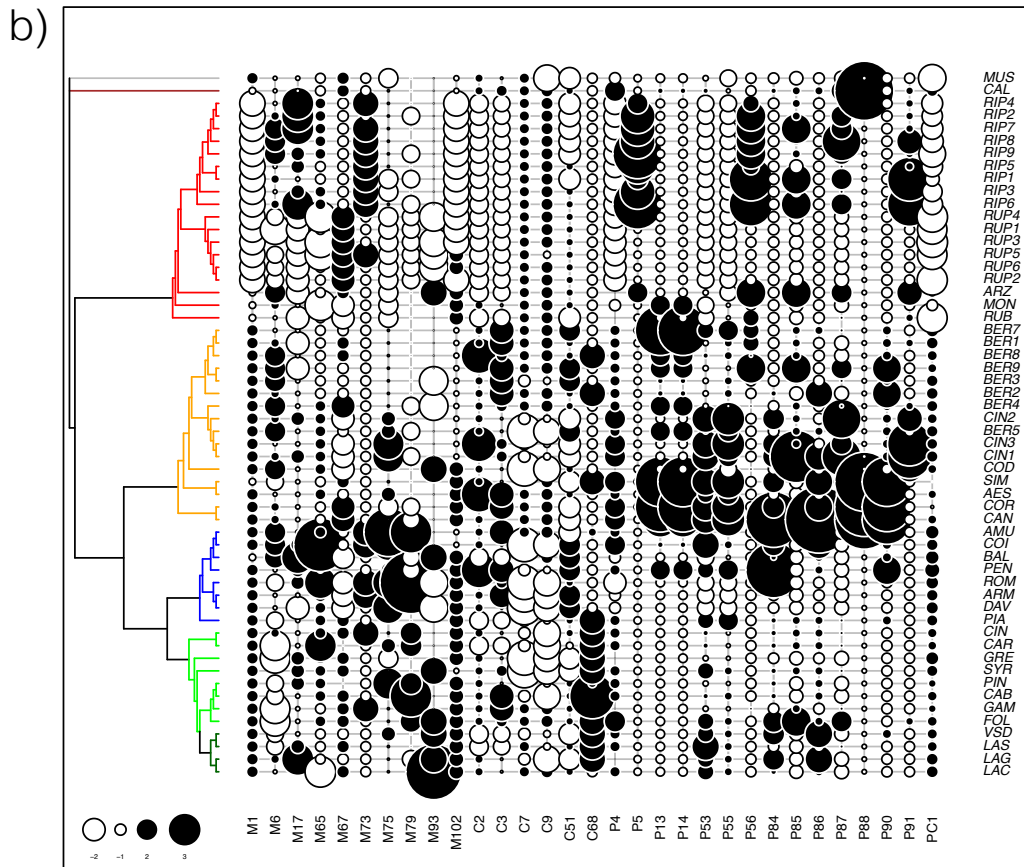

Supplement: S8 Fig — a) leaf measurements, b) OIV codes. (PDF) [file pone.0283324.s008.pdf]
