# Supplementary material for: Species relationships within the genus Vitis based on molecular and morphological data
Source: PLoS One. 2023 Jul 31;18(7):e0283324. doi: 10.1371/journal.pone.0283324 (PMC10389703; doi:10.1371/journal.pone.0283324)
Supplement: S2 Fig — Vitis accessions were grouped in clades and colored as in Fig 1. Wild and cultivated of V. vinifera (EU) are grouped. Known hybrids (CHA, DOA1, LAB), colored in pink, showed high values as well as other accessions that were therefore considered as putative hybrids. (PDF) [file pone.0283324.s002.pdf]

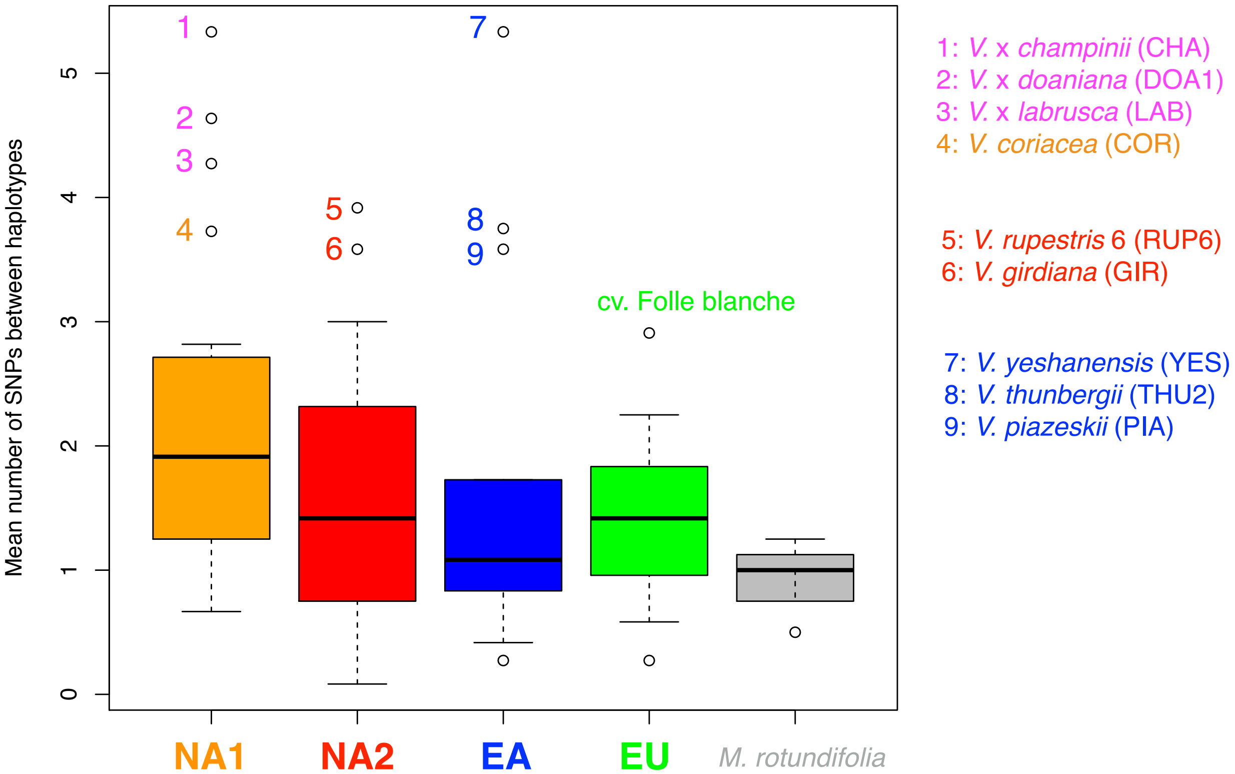

Mean number of SNPs between haplotypes
0
1
2
3
4
5
1
2
3
4
5
6
7
8
9
cv. Folle blanche
NA1
NA2
EA
EU
M. rotundifolia
1: V. x champinii (CHA)
2: V. x doaniana (DOA1)
3: V. x labrusca (LAB)
4: V. coriacea (COR)
5: V. rupestris 6 (RUP6)
6: V. girdiana (GIR)
7: V. yeshanensis (YES)
8: V. thunbergii (THU2)
9: V. piazeskii (PIA)
