# Supplementary material for: Species relationships within the genus Vitis based on molecular and morphological data
Source: PLoS One. 2023 Jul 31;18(7):e0283324. doi: 10.1371/journal.pone.0283324 (PMC10389703; doi:10.1371/journal.pone.0283324)
Supplement: S9 Fig — a) leaf measurements, b) OIV codes. (PDF) [file pone.0283324.s009.pdf]

a)

b)

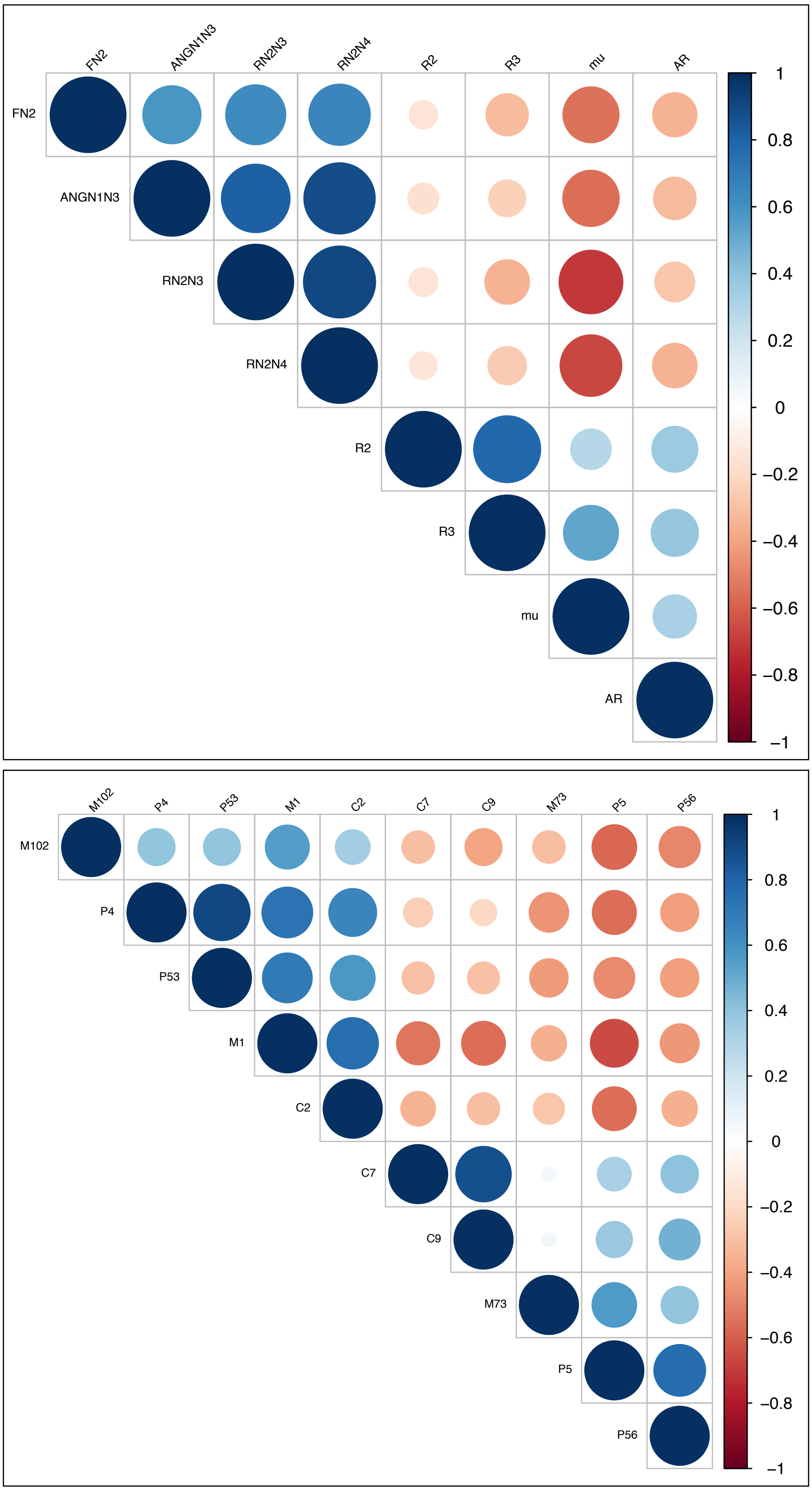

FN2
ANGN1N3
RN2N3
RN2N4
R2
R3
mu
AR
1
0.8
0.6
0.4
0.2
0
−0.2
−0.4
−0.6
−0.8
−1
M102
P4
P53
M1
C2
C7
C9
M73
P5
P56
