# Supplementary material for: Species relationships within the genus Vitis based on molecular and morphological data
Source: PLoS One. 2023 Jul 31;18(7):e0283324. doi: 10.1371/journal.pone.0283324 (PMC10389703; doi:10.1371/journal.pone.0283324)
Supplement: S2 Table — (PDF) [file pone.0283324.s011.pdf]

S2 Table. List of loci sequenced.

Locus	Forward primer	Reverse primer	Size (pb)[a]	N°Chr.	Position_start[b]	Position_end[b]
GAI	ATGGATGAGCTTCTCGCTGT	TAGAAGTGCATCTGGAGAAT	650	1	4896335	4897079
TFL1	CATACGCACATAGGAAAGGC	GAGTCCCAGAGGTCAACTT	1330	6	21338450	21337001
LDOX	TTTAGCCCAATCATATTAGTTCC	TAAAAGAGAGCGCGTCTCACTAG	260	8	7600719	7601079
CHI1	ACCAAAGCTCGTACAAGGACA	TGGCAGCAGATGACAAATATG	703	13	1556565	1555765
DFR4	GCTGACAGATTTGGGGTTTGA	CCCTATGCAAACACAAACGA	219	18	10907687	10907322
TC1-A	GATTCTCCATTGTATGACTTG	TCAGAATGAAGCACAACACAAA	312	5	4472329	4472628
TC1-B	AGGTGCACATCATCCATCAA	TTACCATGGGGAAGTCAAGC	323	5	4472826	4473421
255A	CTTGTTGTTTTAGCGGAG	TAAGGGTGGTAGTTTCATC	369	18	3538551	3539887
1526A	ATTACCAGAATGAAGTAGAAG	GAAGTAGGGGTGTGTCTC	526	3	669042	670330
2351A	TACGAGAAGGAGTGTTGTC	ATTATTCCCAAACCTACC	704	17	1162378	1163535
4194A	GAGTGGGTTCTCAAGTTC	TATTTAGCAGGTTCTTTACC	617	13	569164	570262
4275A	CGTTGAAGTCGGTTTATC	GAAGTAGGGCGTAGTCTGT	758	9	21917114	21918459
Total			6771			

[a]Final size in base pairs (bp) within the alignment

[b]Position on the reference genome of *Vitis vinifera*
