# Supplementary material for: Species relationships within the genus Vitis based on molecular and morphological data
Source: PLoS One. 2023 Jul 31;18(7):e0283324. doi: 10.1371/journal.pone.0283324 (PMC10389703; doi:10.1371/journal.pone.0283324)
Supplement: S3 Table — (PDF) [file pone.0283324.s012.pdf]

S3 Table. SNP numbers in the array according to the discovery panel.

Species	Nb individuals	Nb SNP in array	Nb SNP assayed	Nb SNP analyzed (%)
V. vinifera* subsp. *vinifera	44	15,022*	13,375	10,774 (83.1)
V. vinifera* subsp. *sylvestris	5			
V. aestivalis	5	1,000	912	464 (3.6)
V. lincecumii	1	400	354	188 (1.4)
V. cinerea* var. *berlandieri	3	1,000	913	409 (3.2)
V. cinerea* var. *cinerea	3	1,000	905	365 (2.8)
V. labrusca	1	1,000	905	437 (3.4)
V. labrusca* x *vinifera	2			
Muscadinia rotundifolia	1	578	520	334 (2.6)
**Total**	65	20,000	17,884	12,971(100)

* included 24 SNP cpDNA + 163 SNP vinifera spain ?
