# Supplementary material for: Species relationships within the genus Vitis based on molecular and morphological data
Source: PLoS One. 2023 Jul 31;18(7):e0283324. doi: 10.1371/journal.pone.0283324 (PMC10389703; doi:10.1371/journal.pone.0283324)
Supplement: S4 Table — (PDF) [file pone.0283324.s013.pdf]

S4 Table. Description of leaf measurements and their phylogenetic signal

Var_names1[a]	Var_names2[b]	Type	Description	A_pvalue[c]
LU	l	distance	leaf length	0.003**
LA	w	distance	leaf width	0.048*
LUxLA	l x w	dist x dist	product of leaf length and width	0.042*
ON1	L_1	distance	length of vein N1	0.002**
N2N2'		distance	distance between N2 and N2' extremities	0.012**
N3N3'		distance	distance between N3 and N3' extremities	0.069[ns]
N4N4'		distance	distance between N4 and N4' extremities	0.001**
SPSP'		distance	distance between SP and SP' (sinus opening)	0.002**
ON2	L_2	distance[d]	length of vein N2 (between O and extremities)	0.005**
ON3	L_3	distance[d]	length of vein N3 (between O and extremities)	0.055[ns]
O3N4		distance[d]	distance between O3 and N4	0.232[ns]
O4N5		distance[d]	distance between O4 and N5	0.277[ns]
OO3		distance[d]	distance between petiolar sinus base and O3	0.004**
OS	Si_s	distance[d]	distance between superior sinus base and S	0.065[ns]
OI	Si_i	distance[d]	distance between inferior sinus base and I	0.071[ns]
FN2		distance[d]	distance between F and N2 extremity	0.004**
omega(ω)		angle[d]	angle between N1 and N2 (extremities)	0.001**
eta (η)		angle[d]	angle between N2 and N3 (extremities)	0.005**
tau(τ)		angle[d]	angle between N3 and N4 (extremities)	0.245[ns]
ANGN1N4	∡S	angle[d]	sum(omega+eta+tau)	0.001**
ANGN1N3	∡S’	angle[d]	sum(omega+eta)	0.001**
pi (π)		angle	angle of petiolar sinus opening	0.076[ns]
delta (δ)		angle	angle between N1 and N2-N2'	0.025*
lambda (λ)		angle	angle between N1 and S-S'	0.066[ns]
mu (μ)		angle	angle between N1 and I-I'	0.001**
teta (θ)		angle[d]	angle between N3O-N5	0.641[ns]
RN2N3		ratio	ratio between N2N2' and N3N3'	0.001**
RN2N4		ratio	ratio between N2N2' and N4N4'	0.001**
LU:LA	r	ratio	ratio between leaf length and width	0.002**
RS	Si_s/L_2	ratio[d]	ratio between OS and ON2	0.107[ns]
RI	Si_i/L_3	ratio[d]	ratio between OI and ON3	0.144[ns]
R2	A	ratio[d]	ratio between length of N2 and length of N1	0.001**
R3	B	ratio[d]	ratio between length of N3 and length of N1	0.001**
R4	C	ratio[d]	ratio between length of N4 and length of N1	0.002**
R5		ratio[d]	ratio between length of N5 and length of N1	0.579[ns]
OMETOSOI		ratio[d]	ratio sum(omega+eta) and sum (OS+OI)	0.103[ns]
OMETN2N3		ratio[d]	ratio sum(omega+eta) and sum (ON2+ON3)	0.030*
	AR	ratio	ratio between major/minor axis	0.001**
	Circ.	ratio	$4\pi \times (area/perimeter^2)$	0.001**

[a]Variables names according Superampelo software []
[b]Variables names according Chitwood et al []
[c]Pvalue of the Aboutief’s test [67] performed using adephylo [66]. *<0.05. **<0.01
[d]Averaged values of right and left sides of leaves
