# Supplementary material for: Species relationships within the genus Vitis based on molecular and morphological data
Source: PLoS One. 2023 Jul 31;18(7):e0283324. doi: 10.1371/journal.pone.0283324 (PMC10389703; doi:10.1371/journal.pone.0283324)
Supplement: S5 Table — (PDF) [file pone.0283324.s014.pdf]

S5 Table. List and characteristics of OIV codes analyzed.

OIV_code	Organ	Description	Scale	Levels	Type
OIV_001	Young shoot	opening of the shoot tip	1-3-5	closed/half open/fully open	Morphology
OIV_006	Shoot	attitude before tying	1-3-5-7-9	erect/semi-erect/horizontal/semi-drooping/drooping	Morphology
OIV_016	Shoot	number of consecutive tendrils	1-2	2 or less/3 or more	Morphology
OIV_017	Shoot	length of tendrils	1-3-5-7-9	up to about 10 cm/about 15 cm/about 20 cm/about 25 cm/about 30 cm and more	Morphology
OIV_065	Mature leaf	size of blade	1-3-5-7-9	very small/small/medium/large/very large	Morphology
OIV_067	Mature leaf	shape of blade	1-3-5-7-9	cordate/wedge-shaped/pentagonal/circular/kidney/shaped	Morphology
OIV_072	Mature leaf	goffering of blade	1-3-5-7-9	absent or very weak/weak/medium/strong/very strong	Morphology
OIV_073	Mature leaf	undulation of blade between main or lateral veins	1-9	absent/present	Morphology
OIV_074	Mature leaf	profil of blade in cross section	1-2-3-4-5	flat/V-shaped/involute/revolute/twisted	Morphology
OIV_075	Mature leaf	blistering of upper side of blade	1-3-5-7-9	absent or very weak/weak/medium/strong/very strong	Morphology
OIV_079	Mature leaf	overlapping of petiole sinus	1-3-5-7-9	very wide open/open/closed/overlapped/strongly overlapped	Morphology
OIV_093	Mature leaf	length of petiole compared to length of middle vein	1-3-5-7-9	much shorter/slightly shorter/equal/slightly longer/much longer	Morphology
OIV_101	Woody shoot	cross section	1-2-3	circular/elliptic/oblate	Morphology
OIV_102	Woody shoot	relief surface	1-2-3	smooth/ribbed/striate	Morphology
OIV_104	Woody shoot	lenticels	1-9	absent/present	Morphology
OIV_002	Young shoot	distribution of anthocyanin coloration on prostrate hairs of the shoot tip	1-2-3	absent/piping/overall	Color
OIV_003	Young shoot	intensity of anthocyanin coloration on prostrate hairs of the shoot tip	1-3-5-7-9	none or very low/low/medium/high/very high	Color
OIV_007	Shoot	color of the dorsal side of internodes	1-2-3	green/green and red/red	Color
OIV_008	Shoot	color of the ventral side of internodes	1-2-3	green/green and red/red	Color
OIV_009	Shoot	color of the dorsal side of nodes	1-2-3	green/green and red/red	Color
OIV_010	Shoot	color of the ventral side of nodes	1-2-3	green/green and red/red	Color
OIV_015-2	Shoot	intensity of anthocyanin coloration on the bud scales	1-3-5-7-9	none or very low/low/medium/high/very high	Color
OIV_051	Young leaf	color of upper side of blade (4th leaf)	1-2-3-4	green/yellow/bronze/copper-reddish	Color
OIV_068	Mature leaf	number of lobes	1-2-3-4-5	one (entire leaf)/three/five/seven/more than seven	Color
OIV_069	Mature leaf	color of the upper side of blade	3-5-7	pale green/medium green/dark green	Color
OIV_070	Mature leaf	area of anthocyanin coloration of main veins on upper side of blade	1-2-3-4-5	absent/only at the petiolar point/up to the 1st bifurcation/up to the 2nd bifurcation/beyond the 2nd bifurcation	Color
OIV_071	Mature leaf	area of anthocyanin coloration of main veins on lower side of blade	1-2-3-4-5	absent/only at the petiolar point/up to the 1st bifurcation/up to the 2nd bifurcation/beyond the 2nd bifurcation	Color
OIV_103	Woody shoot	main color	1-2-3-4	yellow/brownish/red-violet/grey	Color
OIV_004	Young shoot	density of prostrate hairs on the shoot tip	1-3-5-7-9	none or very low/low/medium/high/very high	Pilosity
OIV_005	Young shoot	density of erect hairs on the shoot tip	1-3-5-7-9	none or very low/low/medium/high/very high	Pilosity
OIV_011	Shoot	density of erect hairs on nodes	1-3-5-7-9	none or very low/low/medium/high/very high	Pilosity
OIV_012	Shoot	density of erect hairs on internodes	1-3-5-7-9	none or very low/low/medium/high/very high	Pilosity
OIV_013	Shoot	density of prostrate hairs on nodes	1-3-5-7-9	none or very low/low/medium/high/very high	Pilosity
OIV_014	Shoot	density of erect hair on internodes	1-3-5-7-9	none or very low/low/medium/high/very high	Pilosity
OIV_053	Young leaf	density of prostrate hairs between main veins on lower side of blade (4th leaf)	1-3-5-7-9	none or very low/low/medium/high/very high	Pilosity
OIV_054	Young leaf	density of erect hairs between main veins on lower side of blade (4th leaf)	1-3-5-7-9	none or very low/low/medium/high/very high	Pilosity
OIV_055	Young leaf	density of prostrate hairs on main veins on lower side of blade (4th leaf)	1-3-5-7-9	none or very low/low/medium/high/very high	Pilosity
OIV_056	Young leaf	density of erect hairs on main veins on lower side of blade (4th leaf)	1-3-5-7-9	none or very low/low/medium/high/very high	Pilosity
OIV_084	Mature leaf	density of prostrate hairs between main veins on lower side of blade	1-3-5-7-9	none or very low/low/medium/high/very high	Pilosity
OIV_085	Mature leaf	density of erect hairs between main veins on lower side of blade	1-3-5-7-9	none or very low/low/medium/high/very high	Pilosity
OIV_086	Mature leaf	density of prostrate hairs on main veins on lower side of blade	1-3-5-7-9	none or very low/low/medium/high/very high	Pilosity
OIV_087	Mature leaf	density of erect hairs on main veins on lower side of blade	1-3-5-7-9	none or very low/low/medium/high/very high	Pilosity
OIV_088	Mature leaf	prostrate hairs on main veins on upper side of blade	1-9	absent/present	Pilosity
OIV_089	Mature leaf	erect hairs on main veins on upper side of blade	1-9	absent/present	Pilosity
OIV_090	Mature leaf	density of prostrate hairs on petiole	1-3-5-7-9	none or very low/low/medium/high/very high	Pilosity
OIV_091	Mature leaf	density of erect hairs on petiole	1-3-5-7-9	none or very low/low/medium/high/very high	Pilosity
