# Supplementary material for: Species relationships within the genus Vitis based on molecular and morphological data
Source: PLoS One. 2023 Jul 31;18(7):e0283324. doi: 10.1371/journal.pone.0283324 (PMC10389703; doi:10.1371/journal.pone.0283324)
Supplement: S6 Table — (PDF) [file pone.0283324.s015.pdf]

S6 Table. List of OIV codes, names used in figures and phylogenetic signal

OIV_code	Type	Var_code	Description	A_Pvalue
OIV_001	Morphology	M1	opening of the shoot tip	0.001**
OIV_006	Morphology	M6	attitude before tying	0.001**
OIV_016	Morphology	M16	number of consecutive tendrils	NA
OIV_017	Morphology	M17	length of tendrils	0.001**
OIV_065	Morphology	M65	size of blade	0.001**
OIV_067	Morphology	M67	shape of blade	0.001**
OIV_072	Morphology	M72	goffering of blade	0.088ns
OIV_073	Morphology	M73	undulation of blade between main or lateral veins	0.002**
OIV_074	Morphology	M74	profil of blade in cross section	0.644ns
OIV_075	Morphology	M75	blistering of upper side of blade	0.001**
OIV_079	Morphology	M79	overlapping of petiole sinus	0.003**
OIV_093	Morphology	M93	length of petiole compared to length of middle vein	0.003**
OIV_101	Morphology	M101	cross section	0.840ns
OIV_102	Morphology	M102	relief surface	0.001**
OIV_104	Morphology	M104	lenticels	0.033*
OIV_002	Color	C2	distribution of anthocyanin coloration on prostrate hairs of the shoot tip	0.001**
OIV_003	Color	C3	intensity of anthocyanin coloration on prostrate hairs of the shoot tip	0.001**
OIV_007	Color	C7	color of the dorsal side of internodes	0.001**
OIV_008	Color	C8	color of the ventral side of internodes	0.934ns
OIV_009	Color	C9	color of the dorsal side of nodes	0.001**
OIV_010	Color	C10	color of the ventral side of nodes	0.674ns
OIV_015-2	Color	C15_02	intensity of anthocyanin coloration on the bud scales	0.091ns
OIV_051	Color	C51	color of upper side of blade (4th leaf)	0.005**
OIV_068	Color	C68	number of lobes	0.001**
OIV_069	Color	C69	color of the upper side of blade	0.01*
OIV_070	Color	C70	area of anthocyanin coloration of main veins on upper side of blade	0.01*
OIV_071	Color	C71	area of anthocyanin coloration of main veins on lower side of blade	0.041*
OIV_103	Color	C103	main color	0.428ns
OIV_004	Pilosity	P4	density of prostrate hairs on the shoot tip	0.001**
OIV_005	Pilosity	P5	density of erect hairs on the shoot tip	0.001**

OIV_011	Pilosity	P11	density of erect hairs on nodes	0.048*
OIV_012	Pilosity	P12	density of erect hairs on internodes	0.048*
OIV_013	Pilosity	P13	density of prostrate hairs on nodes	0.001**
OIV_014	Pilosity	P14	density of erect hair on internodes	0.001**
OIV_053	Pilosity	P53	density of prostrate hairs between main veins on lower side of blade (4th leaf)	0.001**
OIV_054	Pilosity	P54	density of erect hairs between main veins on lower side of blade (4th leaf)	0.022*
OIV_055	Pilosity	P55	density of prostrate hairs on main veins on lower side of blade (4th leaf)	0.001**
OIV_056	Pilosity	P56	density of erect hairs on main veins on lower side of blade (4th leaf)	0.001**
OIV_084	Pilosity	P84	density of prostrate hairs between main veins on lower side of blade	0.007**
OIV_085	Pilosity	P85	density of erect hairs between main veins on lower side of blade	0.006**
OIV_086	Pilosity	P86	density of prostrate hairs on main veins on lower side of blade	0.008**
OIV_087	Pilosity	P87	density of erect hairs on main veins on lower side of blade	0.001**
OIV_088	Pilosity	P88	prostrate hairs on main veins on upper side of blade	0.004**
OIV_089	Pilosity	P89	erect hairs on main veins on upper side of blade	0.065ns
OIV_090	Pilosity	P90	density of prostrate hairs on petiole	0.001**
OIV_091	Pilosity	P91	density of erect hairs on petiole	0.001**
