# Supplementary material for: Species relationships within the genus Vitis based on molecular and morphological data
Source: PLoS One. 2023 Jul 31;18(7):e0283324. doi: 10.1371/journal.pone.0283324 (PMC10389703; doi:10.1371/journal.pone.0283324)
Supplement: S1 Data — (ZIP) [file pone.0283324.s016.zip › README.docx]

# Title of Dataset --- Molecular and morphological data analyzed in the paper : Species relationships in the genus Vitis based on molecular and morphological data

## Description of the data and file structure

Sequence data : sequences of the twelve loci sequenced as fasta files with the name locus.fasta. Each sequence in the files is named loci_accession code. The loci are presented in S2 Table and results in Table 1. Accession codes are given in S1 Table Sequences were concaneted and the final aligment used to perform a Maximum Likehood phylogenetic analysis using MEGA7. Missing position are indicated with « ? ».

Array nuclear data : Nuclear data are provided in the excel file SNP_Array_results.xlsx. The file has three sheets. The first sheet gives the position of SNPs in the reference sequence, the allelic forms and allele frequencies. The second sheet gives the results with SNP in the first column (ordered by chromosome and position along the chromosome). Line 1 (accession code) and line 2 (accession name) both are provided in in S1 Table). The third sheet corresponds to the data frame analyzed with SNP in lines and accessions in column. This table is transposed in the first step in the analysis to create a R object. Missing data is indicated with « NA ».

Array plastid data : CpDNA data are provided in the file cpDNA_70_23.csv with 70 accessions (coded in S1 Table) in lines and 23 SNPs in lines. Missing data is indicated with « NA ».

Leaf measurements : The file Ampelo_56_42.csv gives the measurements of 42 leaf characters obtained with SuperAmpelo (Solvadini et al) in columns for 56 accessions in lines. List of characters is provided in S4 table (and figured in S1 Fig). Accessions codes are given in S1 Table. Measurements in mm, mm2 and degrees.

OIV descriptors : The file OIV_56_46.csv gives records for 56 accessions (coded in S1 Table) for 46 OIV descriptors given in S5 table. Coding of descriptors uses different discrete scales (e.g. 1-2-3, 1-3-5-7-9)
