# Supplementary material for: Species relationships within the genus Vitis based on molecular and morphological data
Source: PLoS One. 2023 Jul 31;18(7):e0283324. doi: 10.1371/journal.pone.0283324 (PMC10389703; doi:10.1371/journal.pone.0283324)
Supplement: S7 Fig — (PDF) [file pone.0283324.s007.pdf]

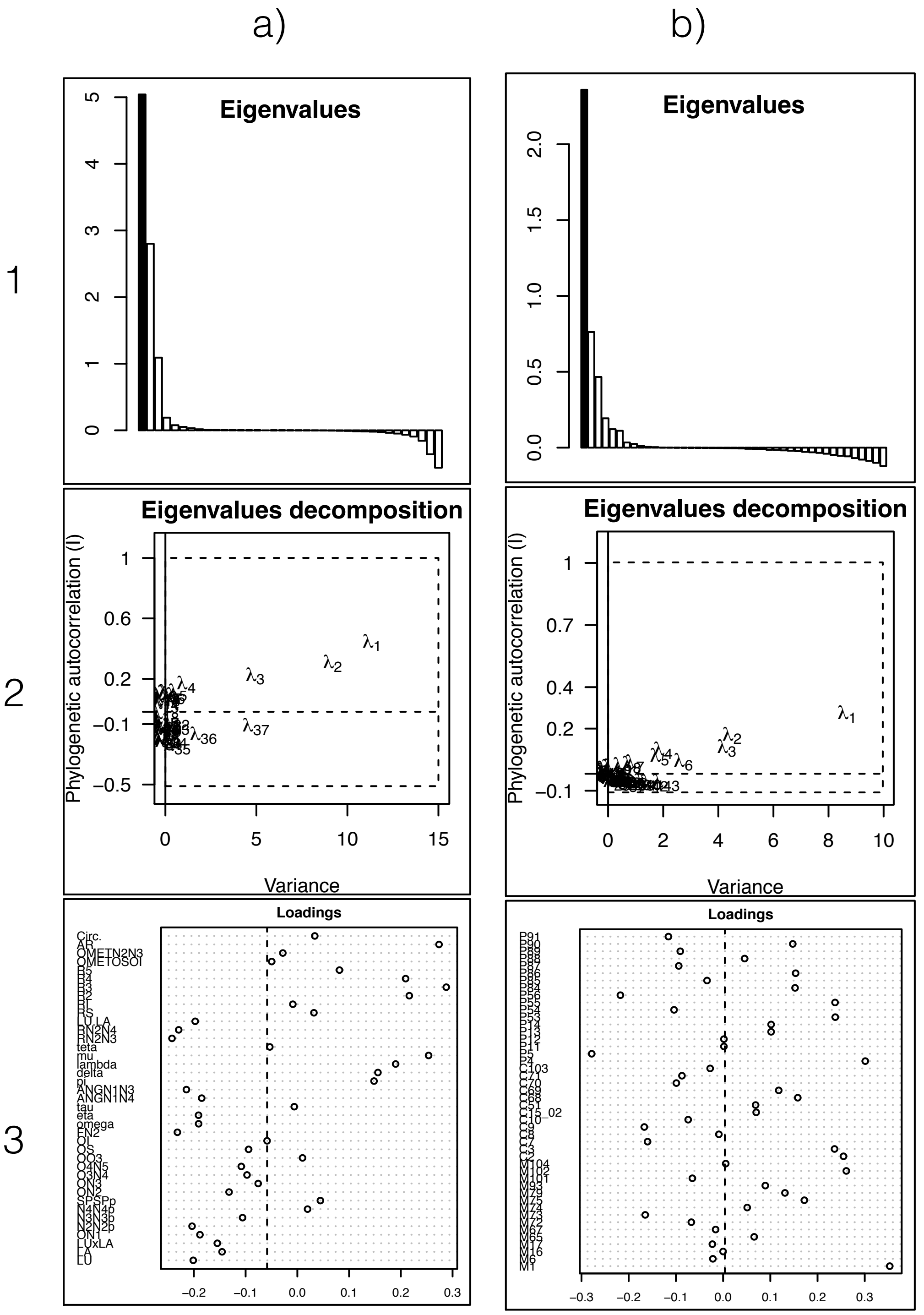
a)
b)
1
2
3
Eigenvalues
Eigenvalues
Eigenvalues decomposition
Eigenvalues decomposition
Phylogenetic autocorrelation (I)
Phylogenetic autocorrelation (I)
Variance
Variance
Loadings
Loadings
